# Single-cell transcriptomic profiling reveals the heterogeneity of epithelial cells in lung adenocarcinoma lymph node metastasis and develops a prognostic signature

**DOI:** 10.3389/fimmu.2025.1637625

**Published:** 2025-07-25

**Authors:** Qiuqiao Mu, Han Zhang, Ying Shi, Mengli Xue, Jingxian Wang, Yun Ding, Lin Tan, Hui Yuan, Xin Li, Daqiang Sun

**Affiliations:** ^1^ Clinical School of Thoracic, Tianjin Medical University, Tianjin, China; ^2^ Department of Thoracic Surgery, Tianjin Chest Hospital, Tianjin, China; ^3^ Haihe Laboratory of Cell Ecosystem, Tianjin, China; ^4^ Chest Hospital, Tianjin University, Tianjin, China; ^5^ Department of Thoracic Surgery, Fujian Provincial Hospital Affiliated to Fuzhou University, Fuzhou, Fujian, China; ^6^ Qingdao Hospital, University of Health and Rehabilitation Sciences (Qingdao Municipal Hospital), Qingdao, China

**Keywords:** LUAD, lymph node metastasis, epithelial cell, SELENBP1, immunotherapy

## Abstract

**Background:**

Lymph node metastasis markedly worsens prognosis in lung adenocarcinoma (LUAD); however, the evolutionary dynamics and regulatory mechanisms underlying the heterogeneity of malignant epithelial cells during this process remain poorly understood and warrant comprehensive investigation.

**Methods:**

We performed a comprehensive single-cell transcriptomic analysis of epithelial cells from 18 samples comprising normal lung tissue and lymph node metastases. Malignant epithelial cells were identified via inferred copy number variation (CNV) profiles. Key malignant subpopulations were further characterized through trajectory inference, cell–cell communication mapping, gene set variation analysis (GSVA), and reconstruction of transcription factor regulatory networks. To assess clinical relevance, we developed and validated a prognostic model—termed the EAS score—based on the transcriptional signatures of malignant epithelial subsets, using integrated data from multiple TCGA and GEO cohorts. The functional role of the hub gene SELENBP1 was experimentally validated through quantitative PCR (qPCR), Western blotting, immunohistochemistry (IHC), Transwell migration assays, colony formation assays, flow cytometry, ROS quantification, and subcutaneous tumorigenesis assays *in vivo*.

**Results:**

Single-cell transcriptomic analysis identified four distinct malignant epithelial subtypes (Clusters 0–3), each characterized by unique patterns of CNV. Leveraging these defined cellular subpopulations, we constructed a highly accurate model for prognostication in LUAD, enabling reliable classification of patients based on clinical outcomes. Through detailed comparisons between groups with divergent prognostic risks, the study revealed notable differences across the tumor microenvironment (TME), including alterations in pathway activity, gene enrichment distributions, mutation profiles, and anticipated responses to immune checkpoint blockade. In addition, functional validation experiments confirmed that SELENBP1 plays a tumor-suppressive role, further supporting its relevance as a potential intervention target in LUAD.

**Conclusion:**

This research provides insights into the evolutionary complexity and heterogeneity of malignant epithelial populations in lymph node metastatic sites of LUAD. It also presents a scoring system based on prognostic indicators, which serves as a reliable tool for forecasting patient survival outcomes. Moreover, the discovery of SELENBP1 as a candidate tumor suppressor emphasizes its importance in guiding both clinical risk categorization and the design of personalized treatment strategies for individuals classified as high-risk LUAD cases.

## 1 Introduction

Lung cancer remains one of the most prevalent malignancies worldwide and is the leading cause of cancer-related mortality, posing a substantial threat to global public health (1, 2). Histologically, it is classified into small cell lung cancer (SCLC) and non-small cell lung cancer (NSCLC), with the latter accounting for approximately 80%–85% of all cases (3, 4). LUAD accounts for the highest proportion of NSCLC diagnoses, with lung squamous cell carcinoma (LUSC) being the next most prevalent subtype (5, 6). Lymph node metastasis frequently emerges during the early stages of disease progression and represents a principal route of tumor dissemination. It is a key determinant of prognosis, and despite advances in treatment, the five-year survival rate for patients with lymph node involvement remains dismal, at approximately 20%–30% (7). However, the evolutionary heterogeneity and regulatory dynamics of malignant epithelial cells during lymph node metastasis in LUAD remain poorly understood. Notably, few studies have systematically profiled these malignant populations at single-cell resolution within metastatic lesions (8).

The TME is a highly dynamic and intricate ecosystem comprising malignant cells, immune infiltrates, fibroblasts, endothelial cells, extracellular matrix components, and a diverse array of cytokines and signaling molecules (9, 10). Beyond supporting tumor cell survival and proliferation, the TME orchestrates tumor initiation, progression, and metastasis through multilayered intercellular crosstalk. In solid tumors such as LUAD, the extent of immune infiltration, the degree of inflammatory response, and the dynamic crosstalk between malignant cells and adjacent stromal components are increasingly recognized as pivotal factors shaping disease progression and influencing tumor biology (11). As insights into the TME have deepened, research has evolved from focusing on individual cell types to delineating the cooperative networks among multiple cellular and molecular constituents. While immune and stromal elements have traditionally been the primary focus (12–14), the regulatory roles of epithelial cells within the TME have garnered increasing interest. As the origin of most solid tumors, epithelial cells not only drive invasion and dissemination via epithelial–mesenchymal transition (EMT), but also modulate immune recruitment, stromal remodeling, and angiogenesis by secreting cytokines and mediating cell–cell and cell–matrix interactions (15). Furthermore, aberrant intracellular signaling within epithelial cells can reshape the immunological and structural milieu of the TME through interactions with neighboring components. Thus, decoding the heterogeneity of epithelial cells during lymph node metastasis in LUAD is crucial for elucidating tumor pathogenesis, informing personalized therapies, and improving clinical outcomes (10).

Single-cell omics technologies represent a cutting-edge approach characterized by high-throughput capabilities, facilitating comprehensive molecular profiling at the individual cell level. These platforms enable simultaneous analysis of genomic sequences and transcriptional activity within single cells, thereby revealing intricate gene regulatory networks and distinct cellular expression landscapes with unprecedented resolution (5, 16). This approach enables the fine-grained classification of diverse cell populations and facilitates a comprehensive understanding of intratumoral molecular features. It plays a pivotal role in delineating tumor heterogeneity, identifying actionable therapeutic targets and clinical biomarkers, and supporting prognosis assessment and personalized treatment strategies (17). In contrast, bulk RNA sequencing captures average transcriptional signals across mixed cell populations within a tissue, thereby concealing the diversity of individual cell types—especially in tumors, which exhibit extensive cellular heterogeneity. Compared to bulk RNA-seq, single-cell RNA sequencing (scRNA-seq) has uncovered drug-resistant subclones in melanoma (18) and has been employed to predict therapeutic responses, including to immunotherapy (19). When integrated with spatial transcriptomics (20), single-cell technologies offer a spatially resolved, multidimensional view of the tumor microenvironment. The remarkable resolution and high-throughput capabilities of scRNA-seq allow for the identification of fine-scale transcriptional heterogeneity, thereby advancing insights into cancer cell molecular mechanisms and promoting the identification of both prognostic indicators and potential targets for therapeutic intervention (21).

In summary, this study provides the first comprehensive single-cell resolution map of epithelial cell heterogeneity in lymph node metastases of LUAD. Four malignant epithelial subpopulations, defined by distinct copy number variation patterns, were accurately identified. Their evolutionary dynamics and functional states within the tumor microenvironment were delineated through trajectory inference, intercellular communication analysis, and transcriptional regulatory network reconstruction. A robust prognostic scoring system (EAS) was developed based on the transcriptional signatures of key subpopulations, exhibiting strong predictive performance and generalizability across multiple independent cohorts. This model effectively stratifies patients by survival risk and potential benefit from immunotherapy, offering valuable insights for clinical decision-making and personalized treatment strategies. Mechanistically, SELENBP1 was identified and experimentally validated as a putative tumor suppressor that modulates LUAD cell proliferation, migration, oxidative stress, and apoptosis, both *in vitro* and *in vivo*, underscoring its potential as a therapeutic target. Collectively, these findings deepen our understanding of epithelial cell functional heterogeneity in LUAD metastasis and provide a theoretical and experimental framework for high-risk patient stratification, optimization of immunotherapeutic approaches, and the development of novel targeted interventions, highlighting substantial scientific and translational relevance.

## 2 Methods

### 2.1 Data acquisition

ScRNA-seq datasets were obtained from a cohort of 18 LUAD patients who had not received prior treatment. The samples consisted of two distinct tissue types: peripheral normal lung tissues (nLung, n = 11) and metastatic lymph nodes (mLN, n = 7). These sequencing data were retrieved from the Gene Expression Omnibus (GEO) under the accession number GSE131907.

To establish a robust prognostic model, multi-omics data including transcriptomic profiles, clinical parameters, and somatic mutation information of LUAD patients were collected from The Cancer Genome Atlas (TCGA) project via the Genomic Data Commons (GDC) portal. The RNA expression data were normalized using the fragments per kilobase of transcript per million mapped reads (FPKM) methodology. To evaluate the model’s capacity for generalization and predictive accuracy, five publicly available validation datasets—GSE31210, GSE37745, GSE50081, GSE68465, and GSE3141—were further incorporated. These datasets, all obtained from GEO, encompassed both gene expression matrices and associated patient outcome data, enabling comprehensive validation of the proposed prognostic framework.

To investigate potential responses to immunotherapy, Immunophenoscore (IPS) data were retrieved from The Cancer Immunome Atlas (TCIA) (https://tcia.at/patients), which integrates immune-relevant genomic features across diverse cancer types. Higher IPS values are typically indicative of enhanced immune responsiveness and improved therapeutic outcomes.

To further investigate the mechanisms underlying immune evasion in LUAD, we utilized quantitative scores reflecting both immune cell dysfunction and rejection, which were derived from the Tumor Immune Dysfunction and Exclusion (TIDE) (22)(http://tide.dfci.harvard.edu) platform. This integration enabled a comprehensive and systematic assessment of each tumor’s potential to escape immune surveillance, thus providing deeper insight into the immunological landscape associated with LUAD progression.

### 2.2 Processing and quality control of single-cell transcriptomic data

ScRNA-seq data were processed using the Seurat R package (version 4.4.0). Raw expression matrices were converted into Seurat objects, followed by quality control filtering. To ensure high-quality single-cell transcriptomic data, cells were filtered according to stringent quality control criteria. Specifically, cells exhibiting fewer than 300 or greater than 8,500 expressed genes, or showing mitochondrial gene expression that accounted for over 10% of total unique molecular identifiers (UMIs), were excluded from downstream analyses. Subsequently, data normalization and scaling were performed prior to dimensionality analysis. Principal component analysis (PCA) was employed, and the first 20 components extracted were retained for subsequent feature reduction and unsupervised clustering. The clustering step was performed using the “FindClusters” function from the Seurat package. During dimensionality reduction and clustering of distal normal lung tissue samples from 11 LUAD patients (n = 11) and untreated lymph node metastasis samples from 7 individuals (n = 7), the resolution parameter was set to 0.8. Low-dimensional embeddings were generated via Uniform Manifold Approximation and Projection (UMAP) for visualization purposes. Manual annotation of cell identities revealed 12 biologically distinct cell clusters, classified based on established marker gene expression profiles. To identify genes exhibiting differential expression across cellular populations, the Wilcoxon rank-sum test was employed as the statistical method. The analysis adhered to rigorous filtering conditions, including a minimum absolute log_2_ fold change threshold of 1, gene expression presence in no less than 25% of cells within a specified cell group, and an adjusted significance level set below 0.05. These stringent criteria ensured the robustness and biological relevance of the selected differentially expressed genes (DEGs).

### 2.3 Inference of malignant epithelial cells in LUAD lymph node metastases

In order to differentiate malignant epithelial cells from their non-malignant counterparts within lymph node metastatic lesions of LUAD, we utilized the InferCNV computational framework. This tool was employed to predict genome-wide alterations in copy number by leveraging gene expression signals and comparing them against a reference set of normal epithelial cells, which served as the baseline for identifying large-scale CNV events (23). Based on the InferCNV official guidelines, we further applied two stringent filtering criteria to ensure the specificity and robustness of malignant cell identification: (1) the inferred CNV profile of a given epithelial cell had to exhibit a Pearson correlation coefficient greater than 0.15 with the average CNV profile of the top 5% most aneuploid tumor cells; and (2) the cell’s average CNV signal strength (average CNV score) had to exceed 0.25. Epithelial cells meeting both criteria were classified as malignant and selected for downstream analyses. To investigate their dynamic cellular states and potential lineage trajectories, the Monocle2 R package was used to reconstruct pseudotime developmental trajectories and assess differentiation hierarchies within the malignant epithelial population (24).

### 2.4 Pathway and prognostic analysis based on malignant epithelial subclusters

We constructed gene sets based on the marker genes of malignant epithelial subclusters (Cluster 0–3) and applied single-sample gene set enrichment analysis (ssGSEA) to calculate enrichment scores for each sample in the TCGA-LUAD cohort, using the GSVA package (v2.0.7). Kaplan–Meier survival analysis was subsequently conducted to evaluate the prognostic differences among the epithelial subpopulations. To further explore the functional heterogeneity of these malignant epithelial subclusters, 50 hallmark gene sets from the Molecular Signatures Database (MSigDB) were incorporated. Gene Set Variation Analysis (GSVA) was performed on a per-cell basis, and average scores were calculated by cell subpopulation to facilitate functional annotation and comparison at the subcluster level (25, 26). Additionally, the marker genes of Cluster 1 and Cluster 2 were intersected with the differentially expressed genes (DEGs) between tumor and adjacent normal tissues in the TCGA-LUAD dataset to generate a core DEG set. This gene set was subsequently subjected to Gene Ontology (GO) and Kyoto Encyclopedia of Genes and Genomes (KEGG) pathway enrichment analyses using the clusterProfiler package (v4.12.0).

### 2.5 Deciphering cell–cell communication and regulatory networks

To investigate intercellular signaling dynamics, we utilized the CellChat R toolkit to analyze the normalized gene expression matrix from the “RNA” assay in the Seurat object, and quantitatively infer alterations in cell–cell communication patterns (27). In accordance with the standard analytical workflow, CellChatDB was employed as the reference framework for ligand–receptor relationship inference. This resource comprises an expertly compiled collection of documented molecular interaction pairs, enabling systematic identification of communication signals between cell types based on expression profiles. Ligands and receptors with high expression levels were identified within each cell population, and significantly enriched ligand–receptor pairs were further analyzed to construct subtype-specific intercellular communication networks (28).

To explore the underlying transcriptional regulatory landscape, we applied the SCENIC (Single-Cell rEgulatory Network Inference and Clustering) workflow in R to infer transcription factor activity and construct regulatory networks across cell populations (29, 30). This enabled the identification of key transcription factors governing cell fate and subtype-specific functional programs.

### 2.6 Development and evaluation of the epithelial-associated signature prognostic model

To investigate the prognostic significance of epithelial-related genes in LUAD, an initial univariate Cox regression analysis was performed to identify gene candidates correlated with patient survival outcomes. This step enabled the preliminary selection of survival-associated biomarkers for subsequent modeling. Leveraging the selected features, we constructed a robust prognostic signature, referred to as the epithelial-associated signature (EAS), by integrating 10 commonly employed machine learning approaches. These included Random Forest, CoxBoost, Elastic Net, Gradient Boosting Machine (GBM), Lasso, plsRcox, Ridge, StepCox, SuperPC, and Survival-SVM, along with 101 pairwise algorithm combinations. The EAS signature (Epithelial-Associated Signature) specifically refers to a prognostic scoring model constructed from a set of signature genes derived from malignant epithelial subpopulations (Cluster 1 and Cluster 2). It serves as a tool for assessing patient prognosis.

The predictive accuracy of each individual model and combination was quantitatively assessed through receiver operating characteristic (ROC) curve evaluation, where the area under the curve (AUC) was designated as the key metric for performance comparison. All candidate models yielded AUC scores above 0.65, reflecting strong generalizability and predictive robustness across diverse algorithmic settings.

### 2.7 Mutation landscape analysis

To characterize the somatic mutational landscape associated with the prognostic model, we employed the maftools R package to systematically analyze mutation frequency patterns across selected candidate gene sets. Using data from the TCGA-LUAD cohort, patients were stratified into four subgroups based on a combination of median EAS risk score and tumor mutational burden (TMB) levels. Kaplan–Meier survival analysis was then performed to compare overall survival among these subgroups, thereby assessing the prognostic implications of integrating EAS-based risk stratification with mutational burden status.

### 2.8 Immune landscape profiling and therapeutic response estimation across risk groups

To delineate immunological disparities between stratified risk populations, we examined transcriptional profiles related to immune checkpoints as well as major histocompatibility complex (MHC) components within both high- and low-risk subgroups (31). Statistically significant alterations in expression patterns were observed, suggesting differential immunogenic features across prognostic categories.

Furthermore, the IPSmetric was utilized to quantify and compare the immune activity landscape between groups, thereby enabling inference of differential sensitivity to immune-based therapies. To enhance understanding of immune evasion pathways, the TIDE computational framework was employed to approximate the likelihood of immune escape in each subgroup. This approach revealed insights into resistance pathways associated with immune checkpoint therapy within the prognostic context.

In parallel, drug response prediction was conducted using the oncoPredict package in R, enabling a comparative assessment of drug sensitivity across the risk-stratified cohorts. Collectively, these integrative analyses provide a rationale for individualized therapeutic strategies and support the advancement of precision immunotherapy in lung adenocarcinoma.

### 2.9 Analysis of tumor microenvironmental differences

To systematically evaluate immune cell composition across prognostic risk groups, seven immune cell infiltration inference algorithms were applied (32). These methods enabled robust quantification of immune landscape variability and provided insights into the differential distribution of immune cell subsets between high- and low-risk groups. To visualize the dynamic changes in immune infiltration, heatmaps were generated to intuitively display the relative abundance of immune cell populations across groups, thereby highlighting potentially relevant immunological differences underlying the risk stratification. To evaluate the immune microenvironment characteristics of LUAD samples, we applied the “estimate” R package to the TCGA-LUAD dataset. This algorithm calculates four scores based on gene expression profiles: Stromal Score, Immune Score, ESTIMATE Score (the sum of stromal and immune scores), and Tumor Purity (inferred from the ESTIMATE Score). We then compared these scores between the high- and low-EAS risk groups to characterize their immune microenvironmental differences.

### 2.10 Tissue acquisition, cell line construction, and ethical approval

Approval for the utilization of human-derived tissue samples was obtained from the Ethics Committee of Tianjin Chest Hospital. Tumorous lesions and their corresponding adjacent non-tumor tissues were collected from individuals undergoing surgical excision for lung adenocarcinoma and were promptly snap-frozen in liquid nitrogen at −80 °C to preserve RNA integrity for downstream experimental applications.

For *in vitro* investigations, a panel of cell lines was employed, comprising one normal human bronchial epithelial line (BEAS-2B) and four well-characterized lung adenocarcinoma lines, including A549, H1299, H1975, and H1650. All cell lines were obtained from the Cell Bank of the Chinese Academy of Sciences (Shanghai, China). These cells were cultured in RPMI-1640 medium (Gibco BRL), enriched with 10% fetal bovine serum (Cell-Box) and 1% penicillin–streptomycin (Biosharp), under standard growth parameters (37 °C, 5% CO_2_, and 95% relative humidity).

To functionally validate the role of SELENBP1, recombinant lentiviral particles harboring the SELENBP1 construct or control vector were obtained from BrainVTA. A549 and H1299 cells were transduced to generate SELENBP1-overexpressing derivatives (A549-SELENBP1 and H1299-SELENBP1), with respective empty-vector controls. Selection of successfully transduced cells was performed using 2 μg/mL puromycin, followed by verification of SELENBP1 upregulation through quantitative real-time PCR and Western blotting.

### 2.11 RNA isolation and quantitative reverse transcription PCR

Total RNA was isolated from cultured cell lines utilizing the UNIQ-10 Column RNA Extraction Kit (Sangon Biotech, B511361) in strict accordance with the supplier’s recommended protocol. First-strand complementary DNA (cDNA) was generated using the gDNA Digester Plus Reagent (Yeasen, HB221101) to eliminate genomic DNA contamination. Quantitative reverse transcription PCR (qRT-PCR) was subsequently conducted with SYBR Green Master Mix (Yeasen, 11141ES) for fluorescence-based amplification detection. GAPDH served as the endogenous reference gene for data normalization. The relative quantification of mRNA expression levels was computed using the comparative Ct method (2^−ΔΔCt). All primer pairs were custom-synthesized by Wuhan Servicebio Technology Co., Ltd.

### 2.12 Western blotting and immunohistochemistry

Western blot analysis was carried out under conventional operating procedures. The primary and secondary antibodies used included SELENBP1 (1:1000), GAPDH (1:5000), goat anti-mouse IgG (1:5000), and goat anti-rabbit IgG (1:5000). For immunohistochemical staining, clinical tissue sections were subjected to a series of preparatory steps, including formalin fixation, dehydration, paraffin embedding, and microtome sectioning. The paraffin-embedded slices were then deparaffinized, rehydrated, and treated for antigen retrieval prior to immunostaining. SELENBP1-specific primary antibody was applied at a working dilution of 1:200 to enable signal detection.

### 2.13 Quantification of proliferation through colony formation assays

To evaluate long-term proliferative capacity, cells were seeded into six-well plates at an initial density of 1 × 10³ cells per well and maintained under standard culture conditions for a total of 14 days. Upon completion of incubation, adherent colonies were washed thoroughly with phosphate-buffered saline (PBS), fixed using 4% paraformaldehyde, and subsequently stained with crystal violet solution (Solarbio, China) to enhance visibility. The number and size of colonies formed were assessed microscopically, providing a quantitative measure of proliferative ability.

### 2.14 Characterization of cell migration and invasion *via* transwell-based functional assays

Cellular migratory and invasive behaviors were investigated in LUAD cells using Transwell chamber-based assays. Specifically, H1299 and A549 cell lines were seeded into the upper compartments of Transwell inserts at a seeding density of 1 × 10⁵ cells per well. In invasion assays, membranes were pre-coated with a layer of Matrigel matrix (Corning, USA) to mimic the *in vivo* extracellular environment, whereas uncoated inserts were utilized for migration assays. Following incubation, cells that traversed the membrane barrier were fixed and stained using crystal violet solution (Solarbio, China). Migrated or invaded cells adhering to the lower membrane surface were imaged and counted to quantify their motility and invasive potential.

### 2.15 Flow cytometric assessment of apoptosis

To assess apoptosis, H1299 and A549 cells were harvested after transfection, and both cells and culture supernatants were collected. Cells were gently digested with trypsin lacking EDTA to preserve membrane integrity. Apoptosis was measured using the YF 488-Annexin V/PI apoptosis detection kit (UElandy) following the manufacturer’s protocol. Briefly, cells were resuspended in 100 μL of 1× binding buffer and incubated with 5 μL of Annexin V–FITC and 5 μL of propidium iodide (PI) in the dark at room temperature for 15 minutes. After staining, 400 μL of 1× binding buffer was added, and apoptosis was immediately analyzed by flow cytometry.

### 2.16 Assessment of intracellular ROS accumulation *via* DHE staining

Intracellular levels of ROS were assessed by employing dihydroethidium (DHE) fluorescence staining. A working solution was prepared by dissolving DHE in fresh culture medium to achieve a final concentration of 2 μM. The existing culture medium was replaced with the DHE staining solution, followed by incubation of the cells at 37 °C in the absence of light for 20 minutes. After staining, the cells were rinsed with fresh medium, and the intracellular ROS signal was visualized using a fluorescence microscope. Fluorescence intensity was used as a surrogate indicator of ROS accumulation.

### 2.17 *In vivo tumor*igenesis assay using subcutaneous xenografts

All animal procedures were conducted in accordance with ethical approval from the Animal Ethics Committee of Tianjin Chest Hospital. To assess the tumorigenic capability of cells *in vivo*, A549 cells stably expressing SELENBP1 (via lentiviral transduction) and their respective control counterparts were suspended in concentrated Matrigel and subcutaneously administered into the dorsal flanks of 5-week-old BALB/c nude mice. After approximately 30 days of tumor growth, the animals were euthanized, and xenograft tumors were surgically excised and harvested for further experimental analysis.

### 2.18 Statistical methods and data analysis

Computational analyses related to bioinformatics were carried out using R programming language (version 4.4.0), while experimental data visualization and statistical evaluation were performed with GraphPad Prism and ImageJ software. For datasets conforming to normal distribution assumptions, intergroup comparisons were executed using either the unpaired Student’s t-test or one-way analysis of variance (ANOVA), depending on the number of groups involved. In cases where data exhibited non-normal distribution, the Wilcoxon rank-sum test (for two groups) or Kruskal–Wallis test (for multiple groups) was employed as the appropriate non-parametric alternative. Kaplan–Meier survival curves were generated for survival analysis, and differences between survival distributions were evaluated using the log-rank test. Correlation strength and direction between variables were assessed using Spearman’s rank correlation coefficient. All statistical tests were two-tailed unless otherwise specified. A p-value < 0.05 was considered indicative of statistical significance. Levels of significance were annotated in the figures and results sections as follows: *p < 0.05; **p < 0.01; ***p < 0.001.In this study, different correlation metrics were applied based on the specific analytical purpose. Pearson correlation coefficients were primarily used to assess the linear similarity between inferred CNV profiles of individual cells and the average CNV profile of the top 5% of high-CNV tumor cells, thereby aiding in the identification of malignant cells. In contrast, Spearman’s rank correlation coefficients were employed to evaluate monotonic associations between molecular features and clinical parameters—such as the relationship between EAS risk scores and tumor mutation burden (TMB)—due to the potential non-normal distribution of these variables.

## 3 Results

### 3.1 Single-cell mapping of the LUAD tumor microenvironment

**Figure 1:**
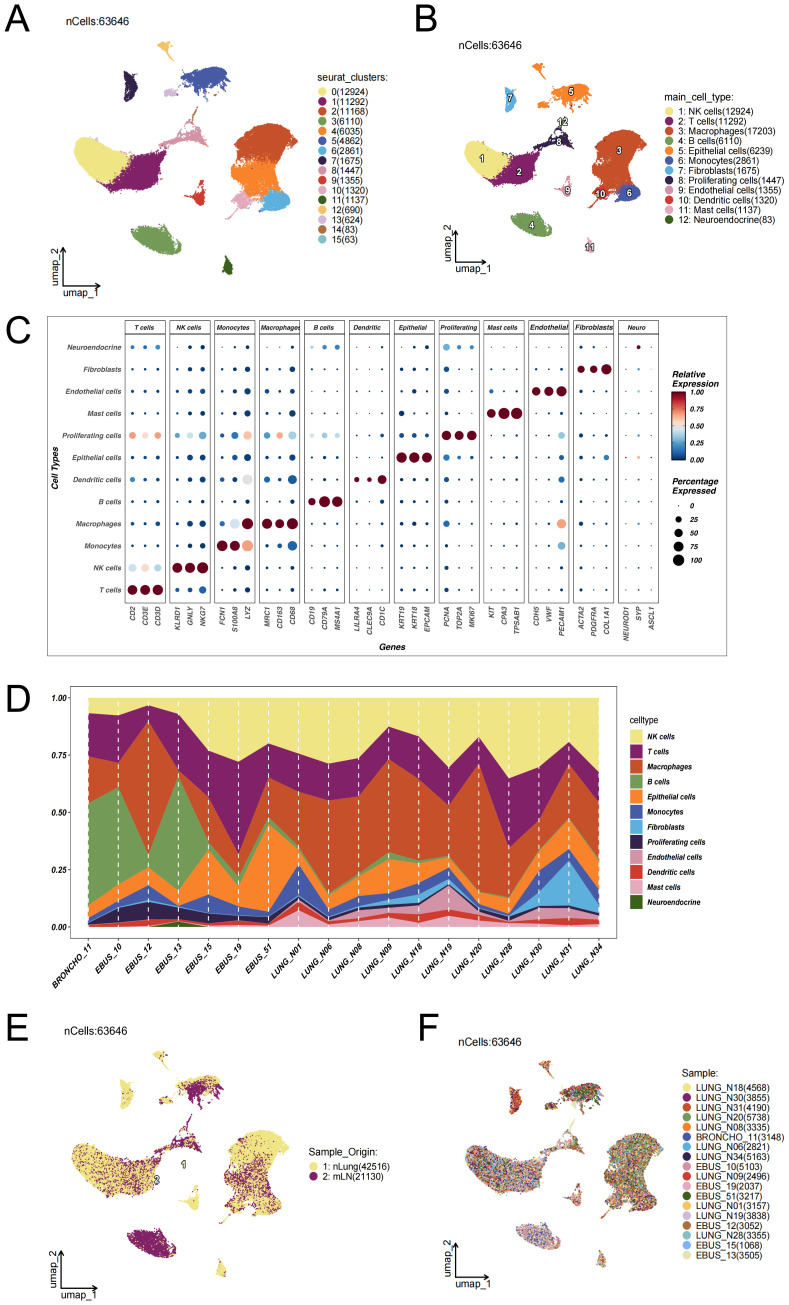
Single-cell transcriptomic landscape reveals the cellular composition and heterogeneity between lung adenocarcinoma lymph node metastases and distal normal lung tissues. **(A)** UMAP dimensionality reduction was performed on 63,646 single cells from 18 samples—including 11 distal normal lung tissues (nLung) and 7 untreated metastatic lymph node (mLN) samples—after rigorous quality control and batch effect correction using the Harmony algorithm (data source: GSE131907). Sixteen transcriptionally distinct cell clusters were identified. **(B)** Based on the expression patterns of canonical marker genes, the clusters were annotated into 12 major cell lineages: epithelial cells, macrophages, T cells, B cells, natural killer (NK) cells, dendritic cells, monocytes, mast cells, fibroblasts, endothelial cells, neuroendocrine cells, and proliferating cells. **(C)** Bubble plot displaying representative marker genes across clusters. The size of each bubble indicates the proportion of cells expressing the gene, while the color intensity reflects the average expression level. **(D)** Proportional distribution of each cell type across the 18 samples. **(E)** Distribution of cells based on tissue origin. UMAP projection of 63,646 cells color-coded by tissue type: yellow for cells from normal lung tissues (nLung, n = 42,516) and purple for cells from metastatic lymph nodes (mLN, n = 21,130). **(F)** Distribution of cells across individual samples. Each color represents a different patient sample.

To investigate epithelial cell heterogeneity in lung adenocarcinoma lymph node metastasis, we analyzed publicly available single-cell RNA sequencing (scRNA-seq) data derived from distal normal lung tissues of 11 LUAD patients (n = 11) and metastatic lymph node samples from 7 untreated individuals (n = 7).After implementing rigorous quality control procedures and correcting for batch effects (**Supplementary Figure 1**), cell identities were assigned by leveraging canonical marker gene signatures, as detailed in the Methods section. Dimensionality reduction via UMAP revealed 16 transcriptionally discrete cellular clusters, forming a high-resolution atlas of cellular heterogeneity (**Figure 1A**). Marker gene expression profiles representative of each cluster were visualized using bubble plots (**Figure 1C**), facilitating the categorization of 63,646 individual cells into 12 distinct cell lineages (**Figure 1B**). These included macrophages, natural killer (NK) cells, T cells, epithelial cells, B cells, monocytes, fibroblasts, proliferative cells, endothelial cells, dendritic cells, mast cells, and neuroendocrine cells. To evaluate variations between samples, **Figure 1D** displays the distribution of cell type frequencies across all 18 tissue specimens. In **Figure 1D**, endothelial cells, mast cells, and fibroblasts appeared more enriched in distal normal lung tissues (nLung), whereas their proportions were relatively lower in lung adenocarcinoma lymph node metastases (mLN). The higher abundance of these cell types in nLung samples suggests a more intact stromal and vascular microenvironment. In contrast, immune cells were more dominant in mLN samples, indicating a relative reduction in stromal-associated cell populations during tumor metastasis. **Figure 1E** illustrates the distribution of cells from different tissue origins in the UMAP space, suggesting notable differences in cellular composition or states between the two sources. **Figure 1F** shows the distribution of cells from individual samples across the entire dataset. The even distribution of cells from each sample among the clusters indicates that batch effects have been effectively eliminated after data integration, providing a solid foundation for subsequent cross-sample comparative analyses.

### 3.2 Inference of malignant epithelial cells in LUAD lymph node metastasis

**Figure 2:**
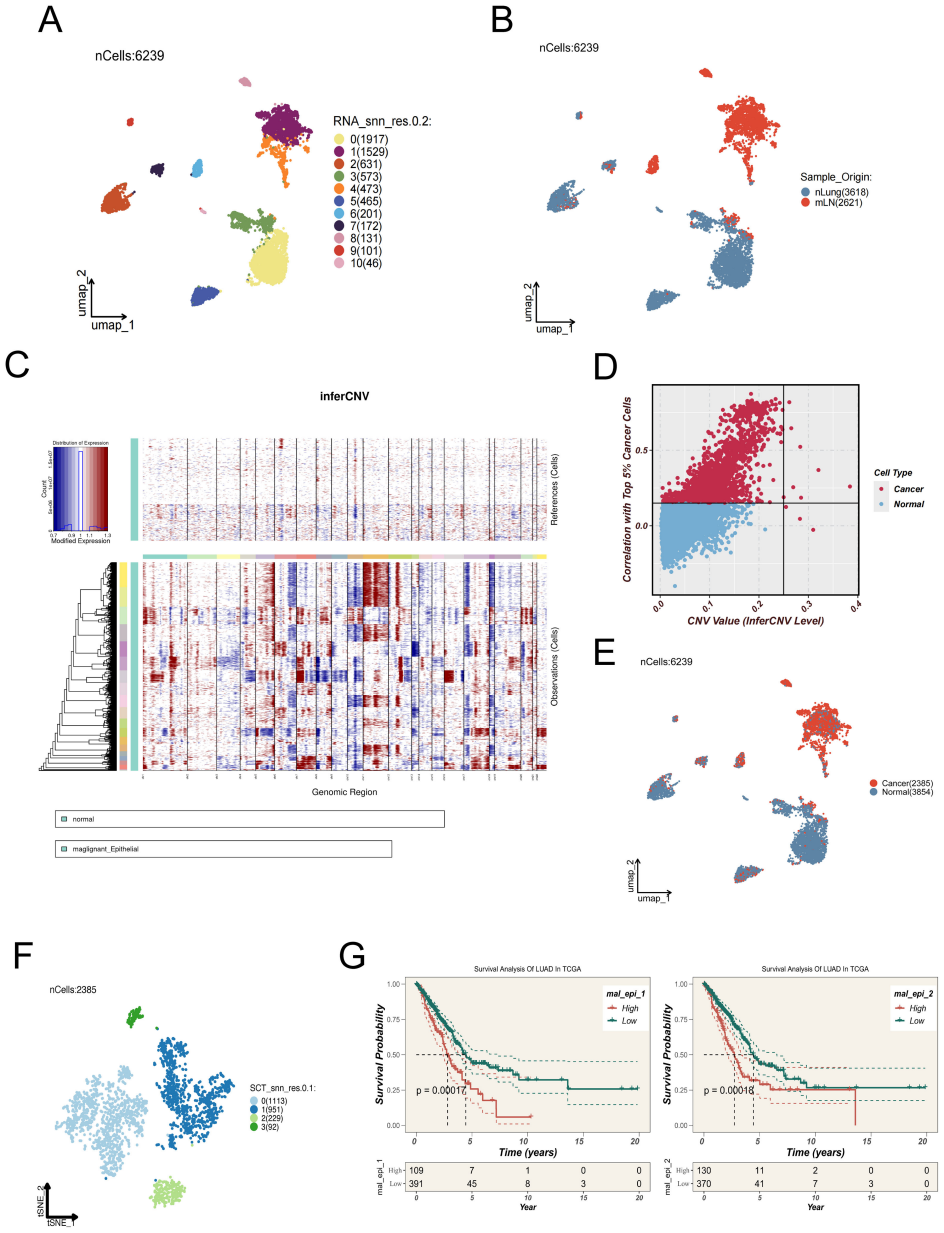
Identification and characterization of malignant epithelial subpopulations in LUAD lymph mode metastases. **(A)** UMAP plot showing 6,239 epithelial cells extracted from distal normal lung tissues and metastatic lymph nodes, which were clustered into 11 distinct subpopulations. **(B)** Spatial distribution of epithelial cells derived from normal lung tissues (nLung, blue) and metastatic lymph nodes (mLN, red). **(C)** Copy number variation (CNV) patterns of epithelial cells were inferred using the inferCNV algorithm, with normal epithelial cells as the reference. The heatmap illustrates regions of chromosomal amplification (red) and deletion (blue) in tumor cells. **(D)** Scatter plot showing the correlation between each epithelial cell and the top 5% of tumor cells with the highest CNV levels (x-axis: CNV value; y-axis: Pearson correlation coefficient). Cells with CNV > 0.25 and correlation > 0.15 were defined as malignant. **(E)** UMAP plot showing the inferred benign (blue) or malignant (red) status of epithelial cells based on inferCNV analysis. **(F)** Malignant epithelial cells were further clustered into four transcriptionally distinct subgroups (Clusters 0–3), which may represent different functional states or differentiation trajectories. **(G)** Signature gene sets for each cluster were constructed and analyzed using ssGSEA in the TCGA-LUAD cohort. Clusters 1 and 2 exhibited higher enrichment scores and were significantly associated with poor survival outcomes, suggesting their potential roles in tumor progression.

To elucidate epithelial cell state transitions associated with lymph node metastasis in LUAD, epithelial cells were extracted from distal normal lung tissues (nLung) and metastatic lymph nodes (mLN) for integrative analysis. Dimensionality reduction and clustering using UMAP revealed distinct spatial organization within the epithelial compartment (**Figure 2A**). Notably, epithelial cells derived from mLN clustered predominantly within a specific region of the UMAP space (**Figure 2B**), suggesting a transcriptomic divergence associated with metastatic transformation.

To determine the malignant potential of epithelial cells, chromosomal CNV analysis was performed using the inferCNV algorithm. In this approach, gene expression profiles from normal epithelial cells served as a reference baseline to infer CNV patterns within tumor samples (**Figure 2C**). Cells were designated as malignant when both their inferred CNV signatures demonstrated a Pearson correlation coefficient greater than 0.15 relative to the mean CNV profile of the top 5% of high-CNV tumor cells, and their CNV signal intensity values exceeded a threshold of 0.25 (**Figure 2D**).A summary of cell classifications derived from inferCNV analysis is presented in **Figure 2E**.

Subsequent UMAP-based visualization and refined clustering of malignant epithelial cells revealed four discrete subclusters (Clusters 0–3; **Figure 2F**), each characterized by distinct transcriptional features. These subgroups may represent different functional states, differentiation trajectories, or levels of metastatic competence. To elucidate the potential functional heterogeneity of the four malignant epithelial subpopulations (Cluster 0–3), we performed GSVA-based pathway enrichment analysis. The results revealed distinct biological features among the clusters: Cluster 0 was predominantly enriched in pathways related to tissue differentiation and endocrine functions, such as MYOGENESIS and PANCREAS_BETA_CELL, suggesting that this group may exhibit tissue-like differentiation or visceral-like characteristics. Cluster 1 showed significant enrichment in cell cycle–associated pathways, including E2F_TARGETS and G2M_CHECKPOINT, indicating high proliferative activity. Cluster 2 was broadly enriched in inflammation, stress response, and metabolic reprogramming pathways, such as COMPLEMENT, HYPOXIA, PI3K_AKT_MTOR_SIGNALING, and UNFOLDED_PROTEIN_RESPONSE, implying that this subgroup may be involved in stress adaptation and immune activation at metastatic sites. In contrast, Cluster 3 was enriched in developmental and stemness-maintaining pathways such as WNT_BETA_CATENIN_SIGNALING and KRAS_SIGNALING_DN, potentially representing an epithelial-plastic or basal-like phenotype. (**Supplementary Figure 3B**).

To explore the potential clinical implications of these subpopulations, single-sample gene set enrichment analysis (ssGSEA) was conducted based on the signature gene expression patterns of each cluster, utilizing data from TCGA LUAD cohorts. Notably, Clusters 1 and 2 exhibited significantly higher enrichment scores, which were correlated with poor clinical outcomes (**Figure 2G**), implying their possible contribution to tumor progression and adverse prognosis.

### 3.3 Pseudotemporal dynamics, intercellular communication, and transcriptional regulation in malignant epithelial cells

**Figure 3:**
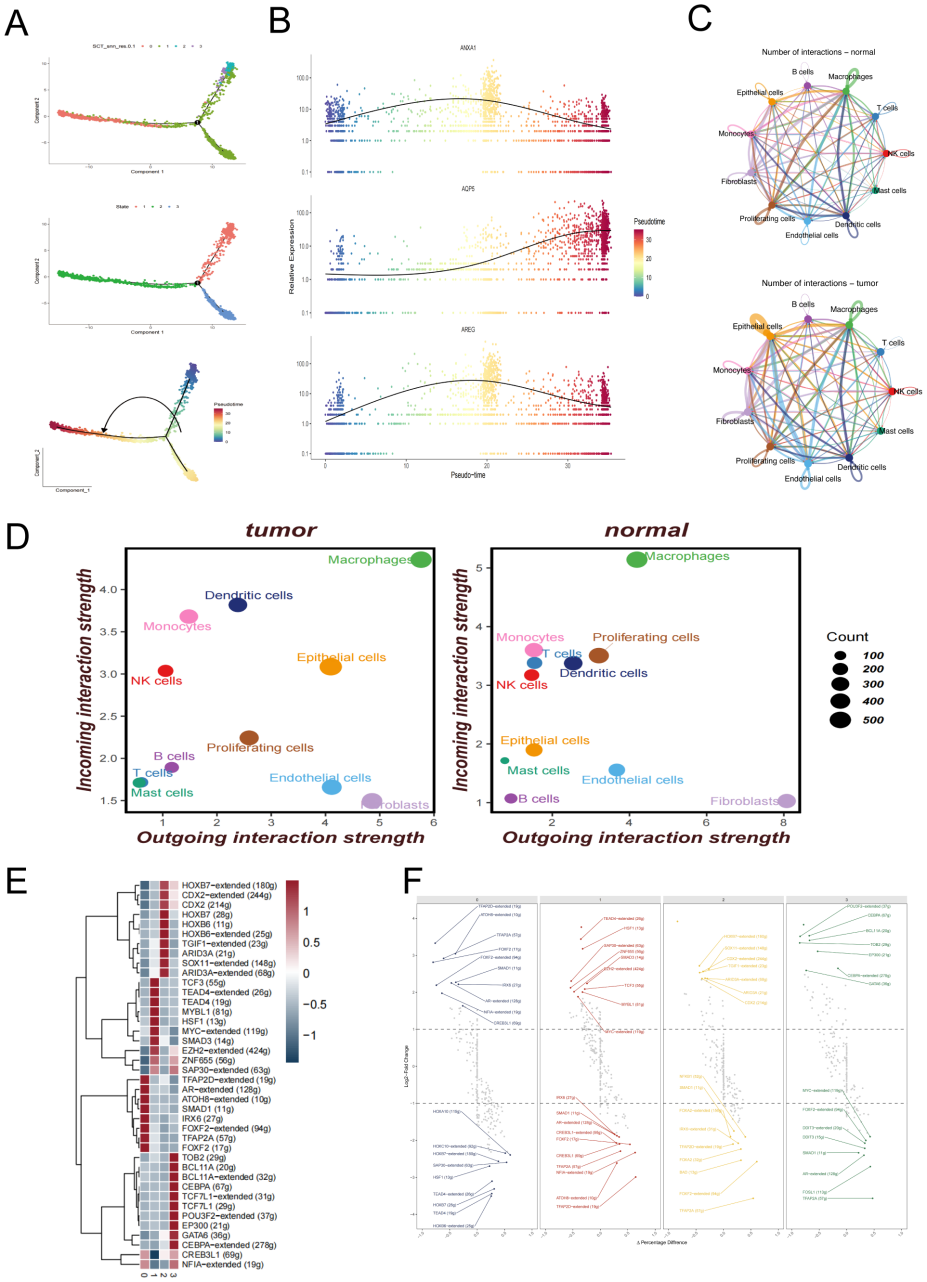
Trajectory inference, cell–cell communication, and transcriptional regulation of malignant epithelial cells. **(A)** Pseudotime trajectory of malignant epithelial cells constructed using Monocle2, illustrating the developmental progression of different subclusters. **(B)** Expression trends of representative genes (ANXA1, AQP5, AREG) along the pseudotime axis. **(C)** Cell–cell communication networks among major cell types inferred by CellChat in normal lung tissues and metastatic lymph node samples. **(D)** Quantitative dot plots showing the strength of incoming and outgoing signals among different cell types in metastatic lymph node and distal normal lung samples. **(E)** Heatmap of differentially expressed transcription factors across malignant epithelial subclusters. **(F)** Visualization of transcription factor regulon activity patterns in each malignant epithelial subcluster.

To explore the dynamic progression of malignant epithelial cells in LUAD lymph node metastases, Monocle2 was employed to infer pseudotime trajectories (**Figure 3A**). The analysis revealed a decline in the proportion of Clusters 2 and 3 along the trajectory, while Clusters 0 and 1 showed increasing trends, suggesting divergent evolutionary paths. Pseudotime-dependent expression dynamics of three representative genes—ANXA1, AQP5, and AREG—are shown in **Figure 3B**, providing insights into key transcriptional shifts across malignant subpopulations (33).

In addition, we performed enrichment analysis of key genes involved in the trajectory differentiation process to clarify the functional divergence between the two cell fate endpoints, as shown in **Supplementary Figure 3C**. Cell fate 1 was significantly enriched in pathways such as cytoplasmic translation and granulocyte chemotaxis, suggesting that this branch may be associated with immune activation or inflammatory responses. In contrast, Cell fate 2 was enriched in pathways including positive regulation of cell adhesion, miRNA catabolic process, and heterotypic cell-cell adhesion, indicating enhanced intercellular adhesion, active transcriptional regulation, and potential EMT-like features.

To further elucidate the functional dynamics along the pseudotime trajectory, we analyzed the progressive transition of cells from early to late states. Cells at the early pseudotime stage were enriched in pathways related to ribosome biogenesis, protein synthesis, and metabolism, reflecting a typically highly proliferative and biosynthetically active status. As pseudotime progressed, Cell fate 1 sustained high levels of protein translation, potentially supporting its acquisition of immune effector functions. Meanwhile, Cell fate 2 became progressively enriched in pathways associated with cell adhesion, transcriptional regulation, and cytoskeletal remodeling, indicating a gradual shift from a metabolically active progenitor state toward a migratory or EMT-like fate.

Cell–cell communication networks were constructed to compare intercellular signaling under normal and metastatic conditions. **Figure 3C** and **Figure 3D** illustrate the ligand–receptor interaction networks among different cell types in normal lung tissues and LUAD lymph node metastases, respectively. Specifically, **Figure 3C** illustrates the overall cell–cell communication networks in both groups. The results reveal markedly enhanced interactions between epithelial cells and immune cells (such as macrophages, T cells, and dendritic cells) in the tumor samples, suggesting a potentially more central regulatory role of epithelial cells within the tumor microenvironment. In addition, endothelial cells exhibited stronger self-interactions as well as increased connections with other cell types, which may reflect tumor-associated angiogenesis and tissue remodeling processes. **Figure 3D** further quantifies these changes using a dot plot, clearly demonstrating that both the number and strength of communications between epithelial cells and macrophages are significantly higher in tumor tissues compared to normal tissues, underscoring their more active role in signal transmission within the tumor immune microenvironment.

To investigate transcriptional regulation within malignant subclusters, **Figure 3E** presents a heatmap of differentially expressed transcription factors, revealing distinct regulatory profiles across the four clusters. Additionally, **Figure 3F** illustrates the differential expression patterns of transcription factor (TF) regulons across the four malignant epithelial subclusters, revealing distinct functional states among them. In Cluster 0, transcription factors such as TFAP2D, FOXK2, and SMAD1 were significantly upregulated, which are associated with epithelial differentiation and anti-proliferative functions. Meanwhile, the downregulation of HOXA10, HOXB7, and TEAD4 further suggests that this subcluster may represent a more differentiated and quiescent state, lacking stem-like or highly proliferative features. In Cluster 1, enriched expression of TEAD4, MYC, and SMAD3—key regulators of cell proliferation, survival, and epithelial–mesenchymal transition (EMT)—along with increased expression of ZFHX2 and TCF3, indicates a highly proliferative, invasive phenotype with enhanced stemness characteristics. Cluster 2 was marked by significant upregulation of CDX2, TGIF1, and ARID3A, suggesting possible lineage reprogramming or activation of WNT-associated transcriptional programs. Conversely, downregulation of FOXA2, BAD, and TFAP2A may reflect suppression of apoptotic pathways and epithelial maintenance mechanisms. In Cluster 3, upregulation of POU3F2, CEBPA, and GATA6 suggests potential involvement in immune modulation and cellular plasticity, while downregulation of MYC, FOXA2, and SMAD1 indicates reduced proliferative activity and a tendency toward immune evasion or a quiescent-like state. Collectively, these results highlight the pronounced transcriptional heterogeneity among malignant epithelial subpopulations and further support their functional divergence within the tumor microenvironment.

### 3.4 Construction and independent validation of a prognostic model for lung adenocarcinoma based on the epithelial-associated signature

Prognostic models play a pivotal role in precision oncology by enabling clinicians to develop individualized treatment strategies based on the molecular characteristics of tumors, while minimizing adverse effects caused by ineffective therapies. Moreover, dynamic risk stratification supports longitudinal disease monitoring and facilitates early detection of recurrence or progression.

In the TCGA-LUAD cohort, we evaluated the enrichment scores of signature genes from four malignant epithelial cell clusters using the ssGSEA method and assessed their association with patient survival. The results showed that high enrichment of Cluster 1 and Cluster 2 was significantly associated with worse prognosis (**Figure 2G**), indicating the potential clinical relevance of these two subpopulations. Based on this, we focused on the marker genes of Cluster 1 and Cluster 2 and intersected them with differentially expressed genes between tumor and adjacent normal tissues in the TCGA cohort, ultimately identifying 65 candidate epithelial-related genes.

**Figure 4:**
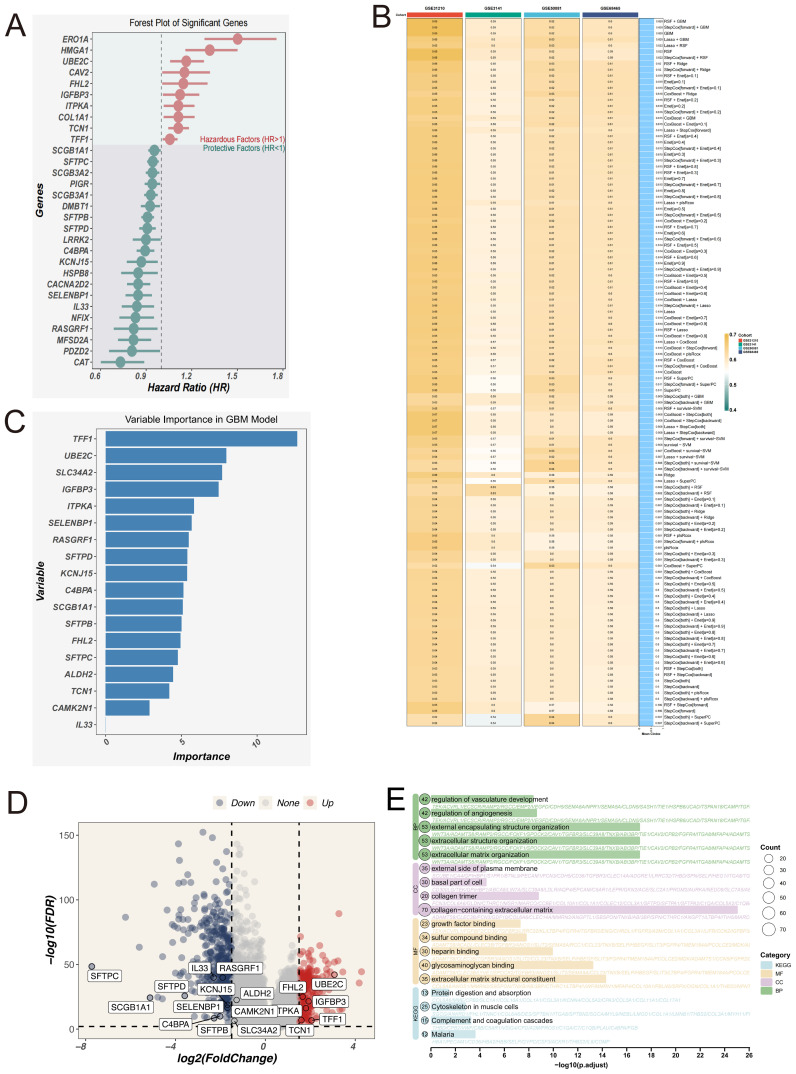
Construction of the EAS scoring model based on signature genes of malignant epithelial cells. **(A)** Forest plot of 30 genes significantly associated with prognosis, identified by univariate Cox regression analysis. **(B)** Heatmap comparing the predictive performance (C-index) of 101 machine learning model combinations—including RSF, GBM, LASSO, CoxBoost, and others—across multiple datasets. **(C)** Feature importance ranking plot from the final GBM model, showing the relative contribution of each gene to the model’s predictive performance. **(D)** Volcano plot illustrating the intersection between differentially expressed genes (normal *vs*. tumor) from the TCGA-LUAD dataset and the signature genes of Clusters 1 and 2. **(E)** GO and KEGG functional enrichment analysis of the intersected genes between differentially expressed genes and the signature genes of Clusters 1 and 2 in the TCGA-LUAD dataset.

We then performed univariate Cox regression analysis on these 65 candidate genes (p < 0.01) and identified 30 genes significantly associated with survival (10 protective and 20 risk-associated), which were visualized in a forest plot (**Figure 4A**), and used to construct the final prognostic model. During the modeling phase, we systematically compared 101 combinations of 10 mainstream machine learning algorithms, including Random Forest, CoxBoost, LASSO, Elastic Net, GBM, and Survival-SVM. Each model was optimized using default internal tuning strategies or grid search functions provided within R packages. Further comparison of different models revealed that the combination of RSF and GBM consistently demonstrated the best performance across all datasets (**Figure 4B**), with an average concordance index (C-index) of 0.626, the highest among all models. Therefore, this method was ultimately selected for constructing the EAS scoring system. The EAS signature (Epithelial-Associated Signature) specifically refers to a prognostic scoring model constructed from a set of signature genes derived from malignant epithelial subpopulations (Cluster 1 and Cluster 2). It serves as a tool for assessing patient prognosis. Feature importance within the model is shown in **Figure 4C**.

We systematically evaluated the predictive performance of the EAS model in comparison with several previously published prognostic models across five independent validation cohorts (TCGA, GSE31210, GSE3141, GSE50081, and GSE68485) (see **Supplementary Figure 3D**).These results demonstrate that the EAS model exhibits strong generalizability and stable prognostic predictive value across multiple independent datasets. By integrating features related to malignant epithelial heterogeneity derived from single-cell transcriptomic data, the EAS model complements existing immune- or epithelium-based prognostic signatures by capturing additional biological information that may otherwise be overlooked. This model thus offers a more biologically interpretable tool for individualized prognostic assessment in LUAD patients.

Differentially expressed genes (DEGs) identified through comparisons between tumor and adjacent normal tissues were cross-referenced with the signature gene sets of Clusters 1 and 2 (**Figure 4D**). Subsequent functional enrichment analysis (**Figure 4E**) revealed significant pathway involvement in angiogenesis regulation, extracellular matrix (ECM) remodeling, ligand-receptor interactions, and integrin-mediated signaling. These results imply that tumor cells may contribute to the metastatic microenvironment by restructuring ECM components and activating pro-metastatic signaling cascades.

**Figure 5:**
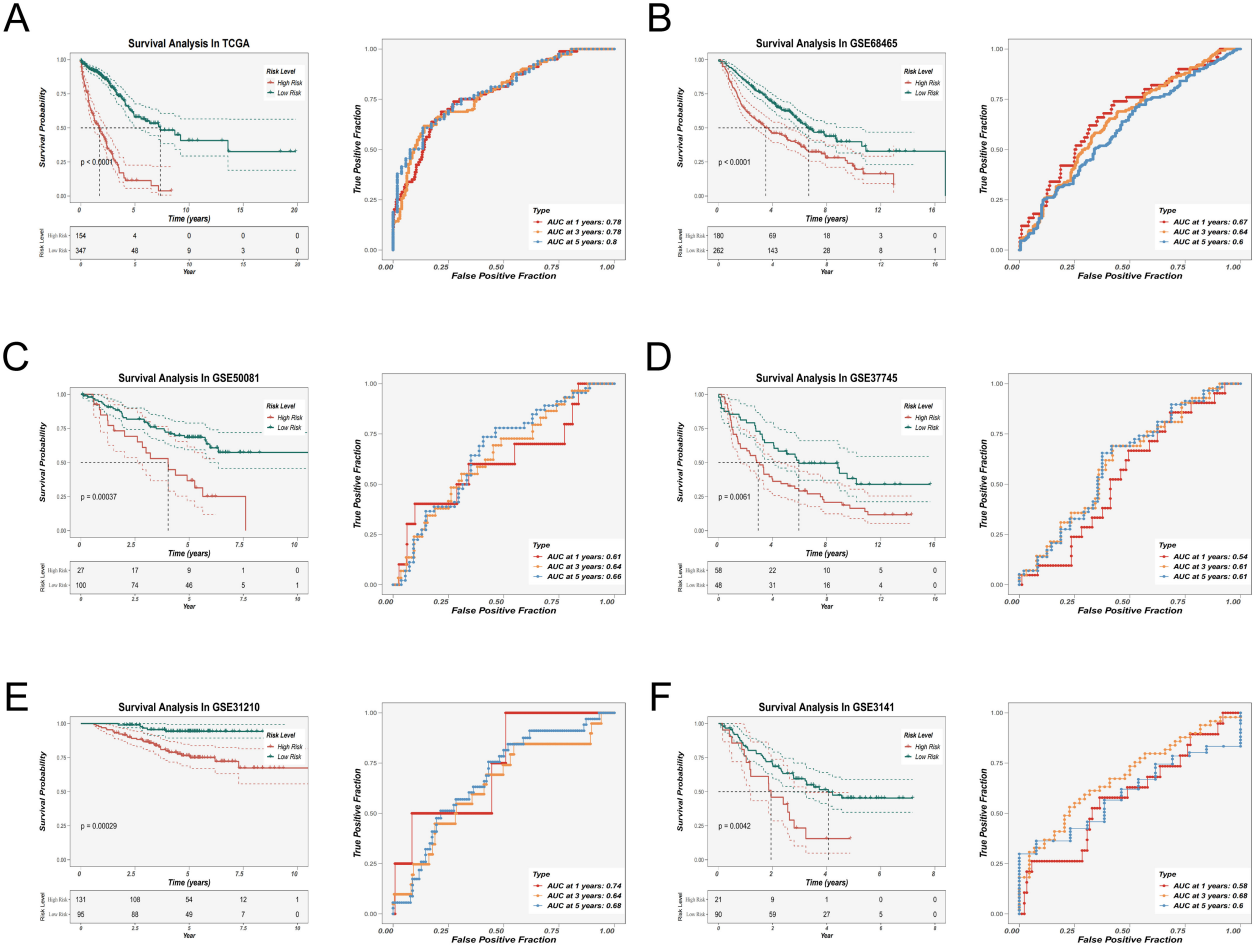
Prognostic evaluation and external validation of the EAS scoring model. **(A–F)** Kaplan–Meier survival analysis and receiver operating characteristic (ROC) curve analysis were performed in the TCGA-LUAD cohort **(A)** and five external GEO validation cohorts **(B–F)** to evaluate the association between the EAS score and overall survival (OS), as well as the prognostic predictive performance of the model.

To evaluate the prognostic significance of the EAS signature, Kaplan–Meier survival analysis was applied, demonstrating that individuals with elevated EAS scores exhibited markedly poorer overall survival in the TCGA LUAD cohort. Additionally, validation using five independent GEO datasets (GSE31210, GSE37745, GSE50081, GSE68465, GSE3141) supported the model’s robustness, generalizability, and reproducibility across diverse clinical populations (**Figure 5A–F**). The prognostic model’s predictive accuracy was further substantiated by receiver operating characteristic (ROC) curve analysis, which confirmed its strong discriminative capacity and clinical relevance in lung adenocarcinoma prognosis. To externally validate the robustness of our model, we analyzed the independent scRNA-seq dataset GSE149655 using the AUCell algorithm. This method assesses the enrichment of model-involved genes in individual cells. The resulting t-SNE visualization (**Supplementary Figure 3A**) revealed that epithelial cells exhibited significantly higher AUCell scores compared to other cell types, indicating active expression of the model gene set. These findings confirm the cell-type specificity and external validity of our model.

### 3.5 Characterization of the tumor immune microenvironment across EAS risk groups

To explore immunological distinctions between patients with high and low EAS scores, a panel of seven distinct computational tools was applied to estimate immune cell infiltration. The resulting profiles were presented in the form of a heatmap (**Supplementary Figure 2A**), highlighting global immune infiltration patterns. Specifically, we found that B cell infiltration levels were consistently higher in the low EAS risk group across multiple immune inference algorithms, including TIMER, CIBERSORT, QUANTISEQ, xCell, and EPIC. Notably, memory B cells also showed an enrichment trend in the low-risk group according to the CIBERSORT and CIBERSORT-ABS tools. In addition, activated mast cells were found to be more abundant in the low-risk group based on both CIBERSORT and CIBERSORT-ABS analyses. The MCPcounter algorithm further indicated stronger infiltration of myeloid dendritic cells in the low-risk group. To deepen this investigation, ssGSEA was implemented to evaluate immune-related pathway activity. The results indicate that, compared to the high-EAS group, the low-EAS group exhibits higher levels of infiltration by various immune cell subsets, particularly dendritic cells (DCs), mast cells, and natural killer (NK) cells. Additionally, enrichment results indicated stronger activation of several immune-associated signaling pathways in the low-EAS group, such as those involved in major histocompatibility complex (HLA) processing and type II interferon (IFN) signaling cascades (**Supplementary Figures 2B, C**). These findings collectively suggest that the low-risk group is characterized by a more immunologically active tumor microenvironment.

To further assess the composition of the immune and stromal compartments, the ESTIMATE algorithm was applied. Correlative analysis identified a significant inverse relationship between the EAS risk score and immune score, while a positive correlation was observed between EAS score and tumor purity (**Supplementary Figure 2D**). A summary of these differences—including stromal score, immune score, ESTIMATE score, and tumor purity—between the high- and low-risk cohorts is provided in **Supplementary Figure 2E**.

### 3.6 Mutation analysis

**Figure 6:**
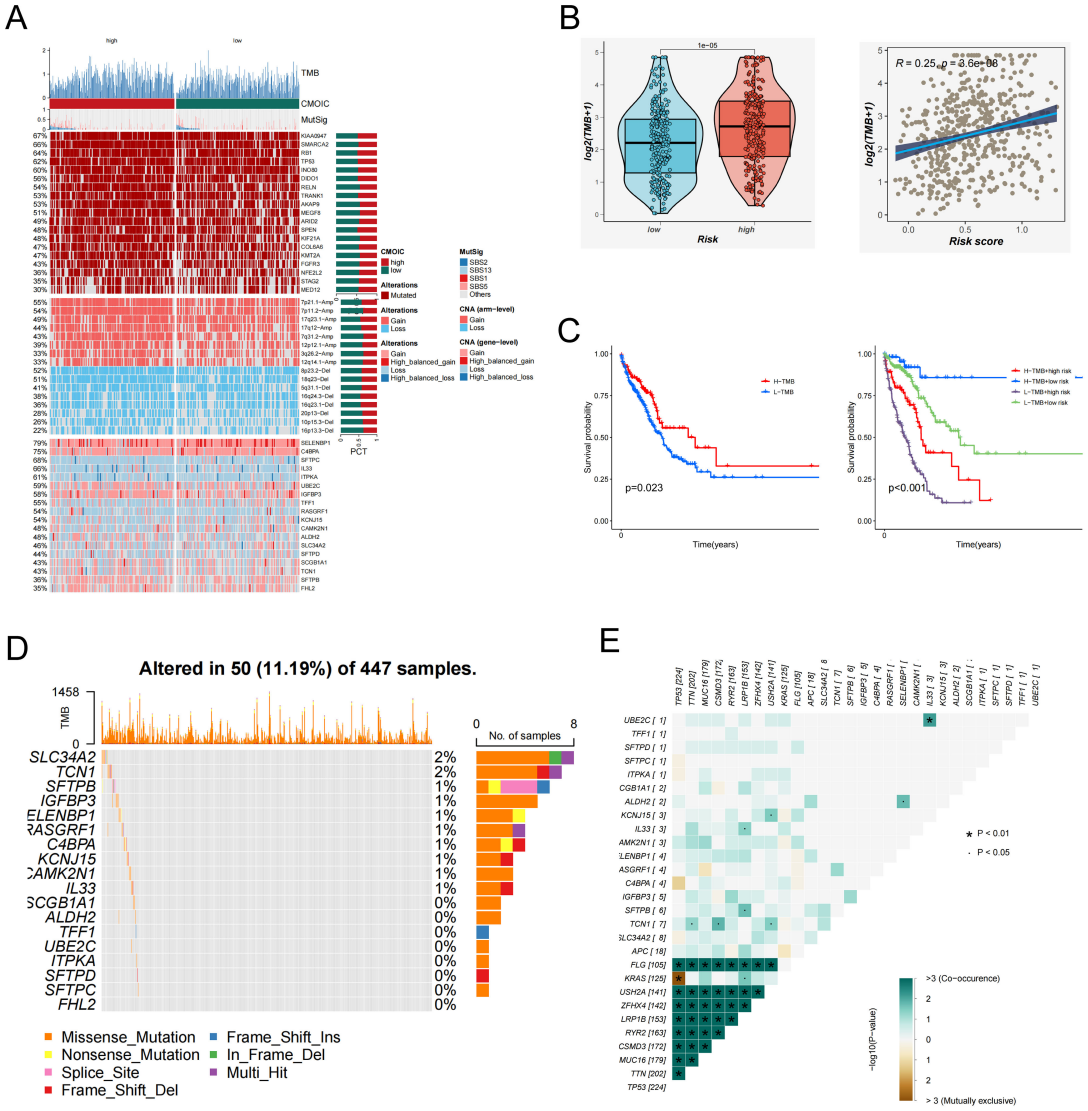
Tumor mutation burden (TMB) and mutation landscape analysis. **(A)** Genomic alteration profiles of patients in the high- and low-EAS score groups. The heatmap illustrates differences in tumor mutational burden (TMB), chromosomal instability (CIN), mutation signatures (MutSig), and other genomic features between the two groups. **(B)** Left: Violin plot comparing the distribution of TMB levels between the high- and low-EAS score groups. Right: Scatter plot showing a positive correlation between EAS scores and TMB levels. **(C)** Left: Kaplan–Meier survival curves comparing overall survival between high and low TMB groups. Right: Integration of EAS scores and TMB levels stratifies patients into four subgroups, illustrating the prognostic impact of different combinations. **(D)** The figure illustrates the mutation frequency and mutation types of EAS-associated core genes in the TCGA-LUAD cohort. **(E)** Co-mutation heatmap depicting the relationships between EAS genes and the top 10 most frequently mutated genes in the cohort. Color intensity indicates statistical significance (−log10 p-value) of co-occurrence or mutual exclusivity.

To investigate the genomic landscape underlying EAS-related risk stratification, tumor mutational burden (TMB) was evaluated across stratified risk cohorts. As illustrated in the heatmap (**Figure 6A**), individuals classified within the high-EAS score subgroup displayed notably increased mutational loads. Quantitative assessments confirmed that TMB levels were significantly elevated in high-risk patients (**Figure 6B**). Furthermore, Spearman’s rank correlation analysis revealed a robust positive correlation between EAS risk scores and TMB values. Elevated TMB has been associated with enhanced neoantigen formation, which can facilitate immune surveillance by increasing tumor antigen visibility and promoting immune-mediated clearance. In line with this notion, Kaplan–Meier survival curves indicated that patients harboring high TMB had substantially improved survival relative to those with lower TMB (**Figure 6C**). When integrating TMB with EAS scores, survival stratification showed that patients with a combination of high EAS score and low TMB had the poorest prognosis, while those with low EAS score and high TMB exhibited the most favorable clinical outcomes (**Figure 6C**), underscoring the synergistic prognostic value of these two variables.

In addition, whole-exome mutation profiling revealed recurrently mutated hub genes, among which SLC34A2 exhibited the highest mutation frequency (**Figure 6D**). Co-mutation network analysis further highlighted patterns of mutational co-occurrence between key hub genes and the ten most frequently mutated genes across the cohort (**Figure 6E**), providing insights into the mutational landscape of LUAD.

Collectively, these findings underscore a complex interplay among EAS risk, tumor mutational burden, and genomic alterations in LUAD, emphasizing the importance of integrative mutation analysis for precise prognostication and therapeutic guidance.

### 3.7 Immunotherapy response evaluation and drug prediction

**Figure 7:**
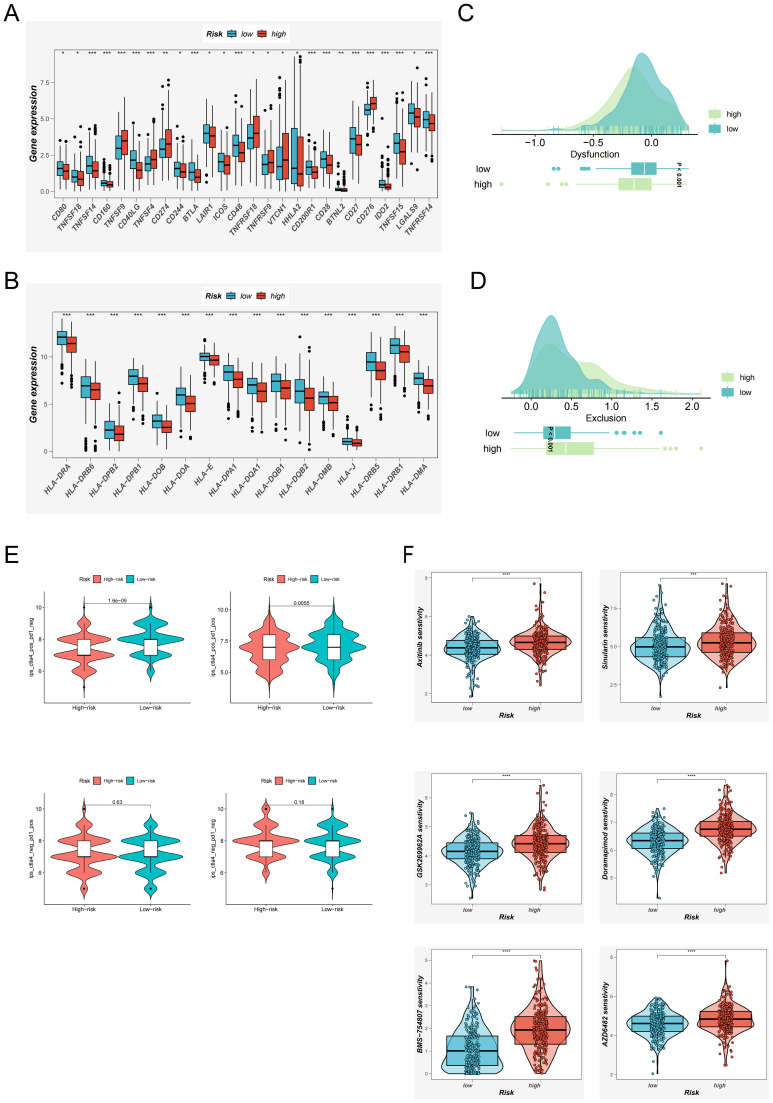
Immunotherapy Response Evaluation and Potential Drug Screening Based on the EAS Score. **(A)** Expression differences of immune checkpoint–related genes between high and low EAS score groups. **(B)** Differential expression of MHC (major histocompatibility complex)–related genes across EAS-defined risk groups. **(C, D)** TIDE analysis evaluating immune dysfunction **(C)** and immune exclusion **(D)** scores to compare functional immune differences between risk groups. **(E)** Immunophenoscore (IPS)–based assessment of potential immunotherapy responsiveness under various immune contexts across EAS risk groups. **(F)** oncoPredict-based analysis of differential drug sensitivity between high and low EAS score groups, identifying candidate compounds with potential therapeutic value. *P < 0.05, **P < 0.01, ***P < 0.001, ****P < 0.0001.

With the expanding clinical adoption of immunotherapy in LUAD, we conducted a comprehensive evaluation of immunotherapeutic responsiveness across EAS-stratified patient cohorts. Transcriptomic profiling revealed that individuals classified into the low-risk group demonstrated significantly elevated expression of the majority of immune checkpoint-associated genes compared to their high-risk counterparts (**Figure 7A**). A comparable expression pattern was also noted for genes related to the major histocompatibility complex (MHC), which were markedly upregulated in the low-risk cohort (**Figure 7B**).

To further elucidate immunological functional differences, we incorporated results from the TIDE framework. This analysis indicated that high-risk patients exhibited a greater propensity for immune escape mechanisms (**Figures 7C, D**), suggesting diminished sensitivity to immunotherapeutic agents. Conversely, IPSassessment highlighted a more favorable immunological landscape among low-risk patients, particularly those exhibiting CTLA-4 positivity. These patients were predicted to derive greater clinical benefit from immune checkpoint inhibition (**Figure 7E**), reinforcing the potential of EAS-based risk stratification in guiding immunotherapy decisions.

Collectively, these findings underscore that the low-risk group is characterized by a more active and responsive immune microenvironment, with higher expression of checkpoint and antigen presentation genes, and greater likelihood of clinical benefit from immunotherapy—particularly in CTLA-4–positive individuals. These results support the utility of risk score–based stratification in guiding immunotherapy decisions and advancing personalized treatment strategies.

In parallel, the oncoPredict R package was used to identify compounds with differential efficacy between risk groups (34). Six agents—Axitinib, Sinularin, BMS-754807, GSK269962A, AZD6482, and Doramapimod—were predicted to exhibit enhanced anti-tumor efficacy in low-EAS patients (**Figure 7F**), highlighting their potential as therapeutic candidates for future clinical development.

### 3.8 Enrichment analysis and immune checkpoints

**Figure 8:**
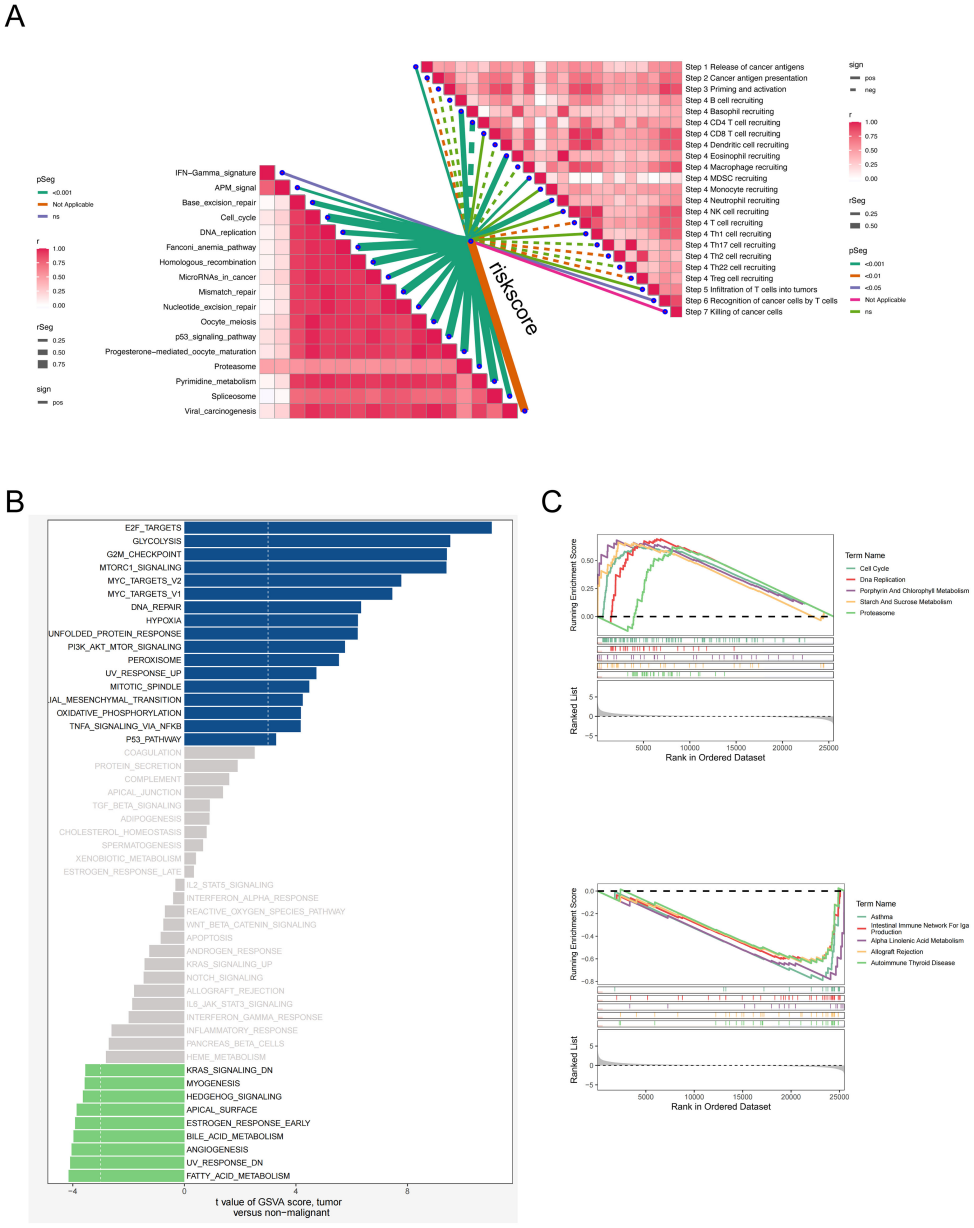
Relationship analysis between EAS score, pathway enrichment, and cancer-immunity cycle. **(A)** Correlation heatmap showing the relationships between the EAS score and different steps of the cancer–immunity cycle as well as hallmark gene sets. **(B)** GSVA-based analysis comparing hallmark pathway enrichment between high and low EAS score groups. **(C)** Gene Set Enrichment Analysis (GSEA) comparing pathway activities between high and low EAS score subgroups.

To better understand the biological processes underlying the EAS scoring system, we comprehensively assessed its associations with hallmark gene sets and the sequential stages of the cancer–immunity cycle. Correlation profiling indicated a prominent inverse relationship between the EAS risk score and most steps of the cancer–immunity cascade, suggesting impaired antitumor immunity in high-risk individuals. In contrast, a majority of hallmark gene pathways exhibited a positive correlation with the EAS score. (**Figure 8A**) Interestingly, elevated EAS scores were also associated with increased recruitment of granulocytic cell populations, including neutrophils and eosinophils, pointing to enhanced granulocyte infiltration within the high-risk subgroup. This may reflect an altered immune microenvironment favoring tumor progression. Additional pathway enrichment analysis revealed that patients in the high-EAS group exhibited upregulation of proliferative and cell cycle-associated signatures—such as those involving E2F targets, glycolytic metabolism, and G2/M checkpoint regulation (**Figure 8B**)—indicative of hyperactive proliferation and energy metabolism. Conversely, the transcriptomic profiles of the low-EAS cohort demonstrated significant enrichment of angiogenesis-related pathways and lipid metabolic processes, hinting at an alternative microenvironmental regulatory state.

Gene Set Enrichment Analysis (GSEA) further confirmed that the high-risk group displayed enhanced activity in pathways associated with cell cycle progression, DNA synthesis, and proteasome-dependent protein degradation, indicative of a highly proliferative and potentially more aggressive tumor phenotype. In contrast, the low-risk group was enriched for immune-dominant pathways, including the IgA immune network and allograft rejection modules, alongside enhanced lipid metabolism (**Figure 8C**). These findings collectively suggest that the low-risk subgroup is characterized by a more immunologically active and metabolically balanced tumor microenvironment, which may impose constraints on tumor expansion and progression.

Collectively, these findings demonstrate that EAS-based risk stratification is tightly linked to tumor cell cycle activity, immune regulation, and metabolic reprogramming—highlighting potential biological targets and therapeutic implications for different LUAD patient subgroups.

### 3.9 Functional validation of SELENBP1 in LUAD *via in vitro* and *in vivo* assays

**Figure 9:**
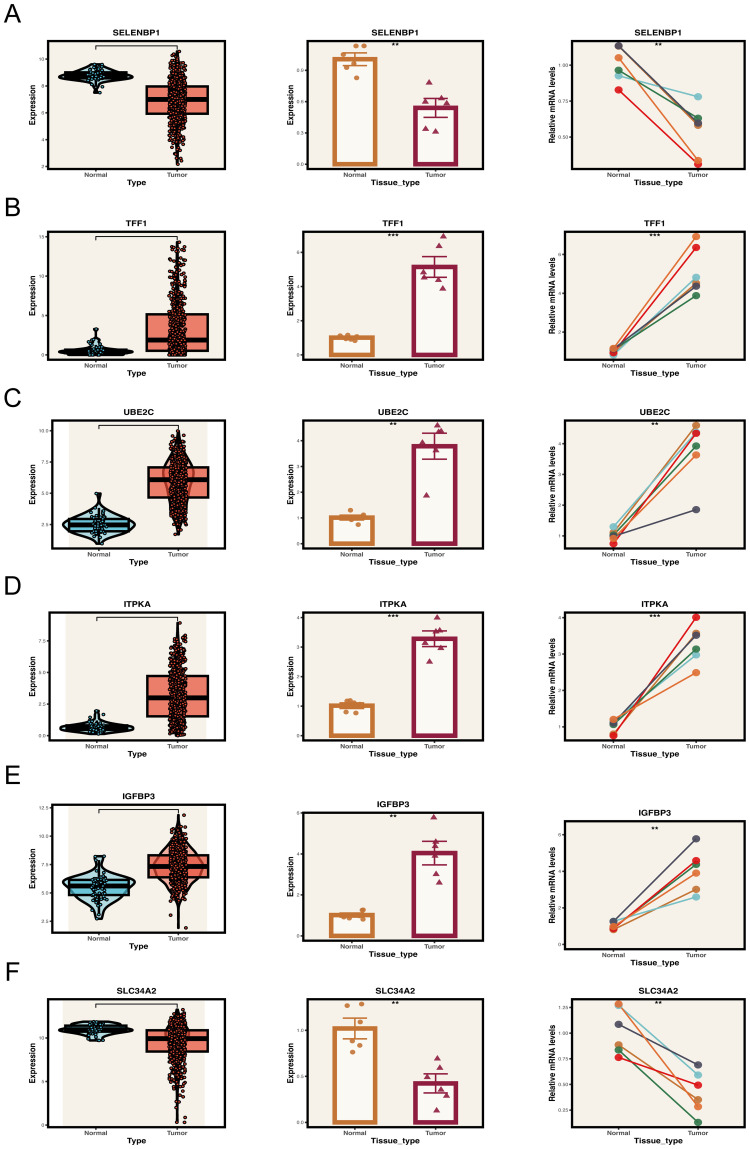
Expression validation of key model genes in clinical samples. **(A–F)** Expression levels of key genes in tumor and normal tissue samples. Left panel: Expression distribution of key genes in the TCGA database. Middle and right panels: Expression of key genes in collected tumor and normal tissue samples. **P < 0.01, ***P < 0.001.

To corroborate the computational predictions generated from publicly available transcriptomic datasets, we carried out a series of experimental validations. Paired tumor and adjacent non-cancerous tissue specimens were obtained from six patients with LUAD who had undergone surgical resection at Tianjin Chest Hospital. Quantitative real-time PCR (qRT-PCR) was employed to experimentally verify the expression patterns of genes associated with the prognostic model. The qRT-PCR results revealed pronounced upregulation of TFF1, ITPKA, UBE2C, and IGFBP3, along with marked downregulation of SLC34A2 and SELENBP1 in tumor samples relative to adjacent normal tissues (**Figures 9A–F**), which was in strong agreement with the transcriptomic profiling outcomes.

**Figure 10:**
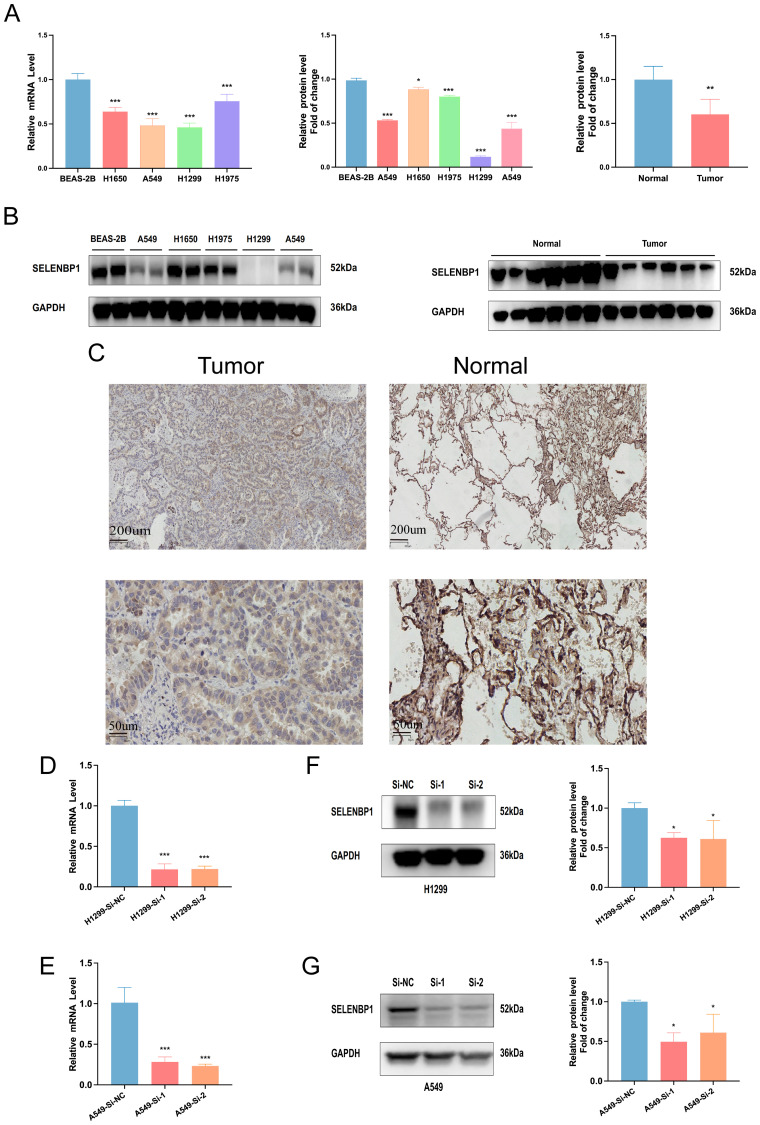
Expression and knockdown validation of SELENBP1 in LUAD tissues and cell lines. **(A, B)** qRT-PCR and Western blotting reveal significantly reduced SELENBP1 expression in four LUAD cell lines (A549, H1650, H1975, and H1299) compared to normal lung epithelial cells (BEAS-2B). **(C)** SELENBP1 expression is significantly decreased in LUAD tumor tissues compared to adjacent normal tissues. **(D, E)** qRT-PCR validation of SELENBP1 siRNA knockdown efficiency in H1299 and A549 cells. **(F, G)** Western blot further confirms decreased protein expression following knockdown. *P < 0.05, **P < 0.01, ***P < 0.001.

Although previous studies have suggested that SELENBP1 may exert tumor-suppressive functions, its role in lung adenocarcinoma (LUAD) has not been systematically elucidated, and the underlying mechanisms remain relatively unclear. In this study, SELENBP1 was identified as a key protective gene within the EAS model. Moreover, based on differential expression analysis and functional enrichment results (**Figure 4E**), SELENBP1 was found to be significantly associated with pathways related to angiogenesis regulation and extracellular matrix (ECM) remodeling, suggesting its potential involvement in the formation and progression of LUAD metastases. Among the candidate genes, SELENBP1 was prioritized for further investigation due to its experimental feasibility, well-established biological rationale, and promising value in both basic and translational research. Expression analysis via qRT-PCR and Western blotting consistently demonstrated that SELENBP1 expression was substantially lower in four widely used LUAD cell lines (A549, H1650, H1975, and H1299) than in the non-tumorigenic bronchial epithelial cell line BEAS-2B (**Figures 10A, B**). Moreover, this pattern of reduced expression was also observed in primary LUAD tumor tissues as compared to their matched non-cancerous counterparts (**Figures 10A–C**), further validating the robustness of the in silico findings.

**Figure 11:**
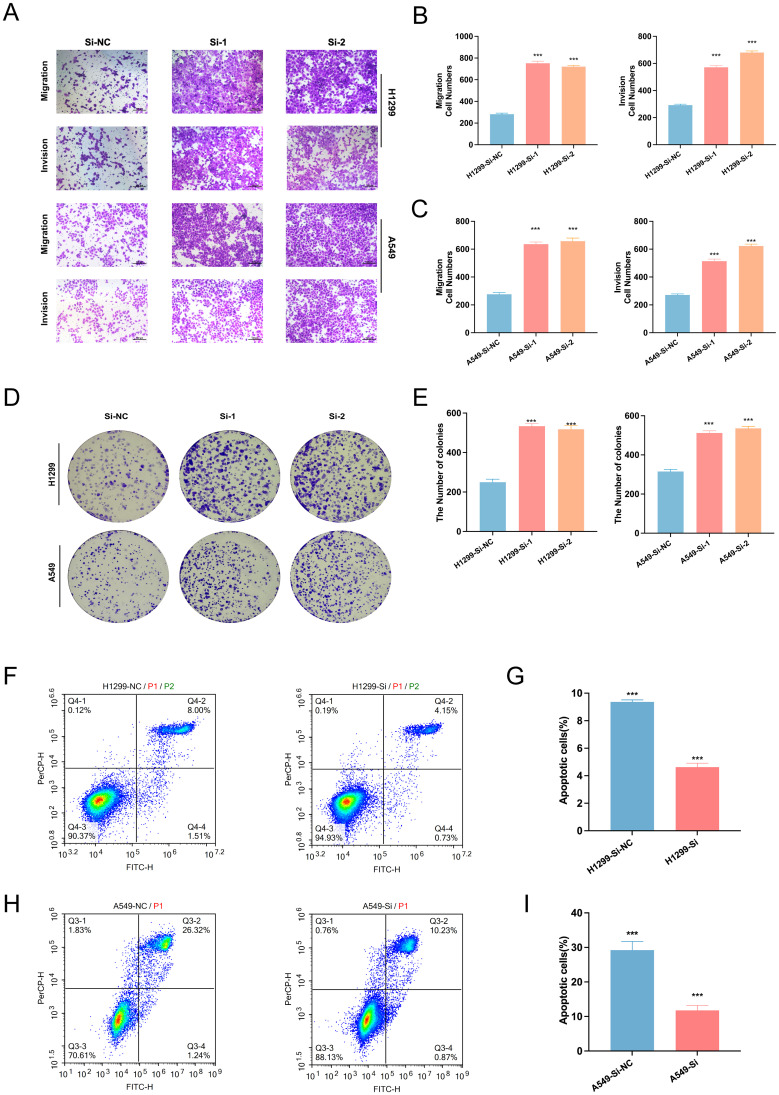
Effects of SELENBP1 knockdown on LUAD cell functions. **(A–C)** Transwell assays demonstrate enhanced migration and invasion capabilities in H1299 and A549 cells following SELENBP1 knockdown. **(D, E)** Colony formation assays show increased clonogenic potential in H1299 and A549 cells after SELENBP1 knockdown. **(F–I)** Flow cytometry analysis indicates a significant reduction in apoptosis in H1299 and A549 cells following SELENBP1 knockdown. ***P < 0.001.

To assess the biological effects of SELENBP1 downregulation, we performed RNA interference using siRNA. Knockdown efficiency was verified by qRT-PCR and Western blotting (**Figures 10D–G**). Functional assays demonstrated that SELENBP1 silencing significantly enhanced LUAD cell migration and proliferation (**Figures 11A–C**), increased colony formation (**Figures 11D, E**), and reduced apoptosis in both A549 and H1299 cells (**Figures 11F–I**).

**Figure 12:**
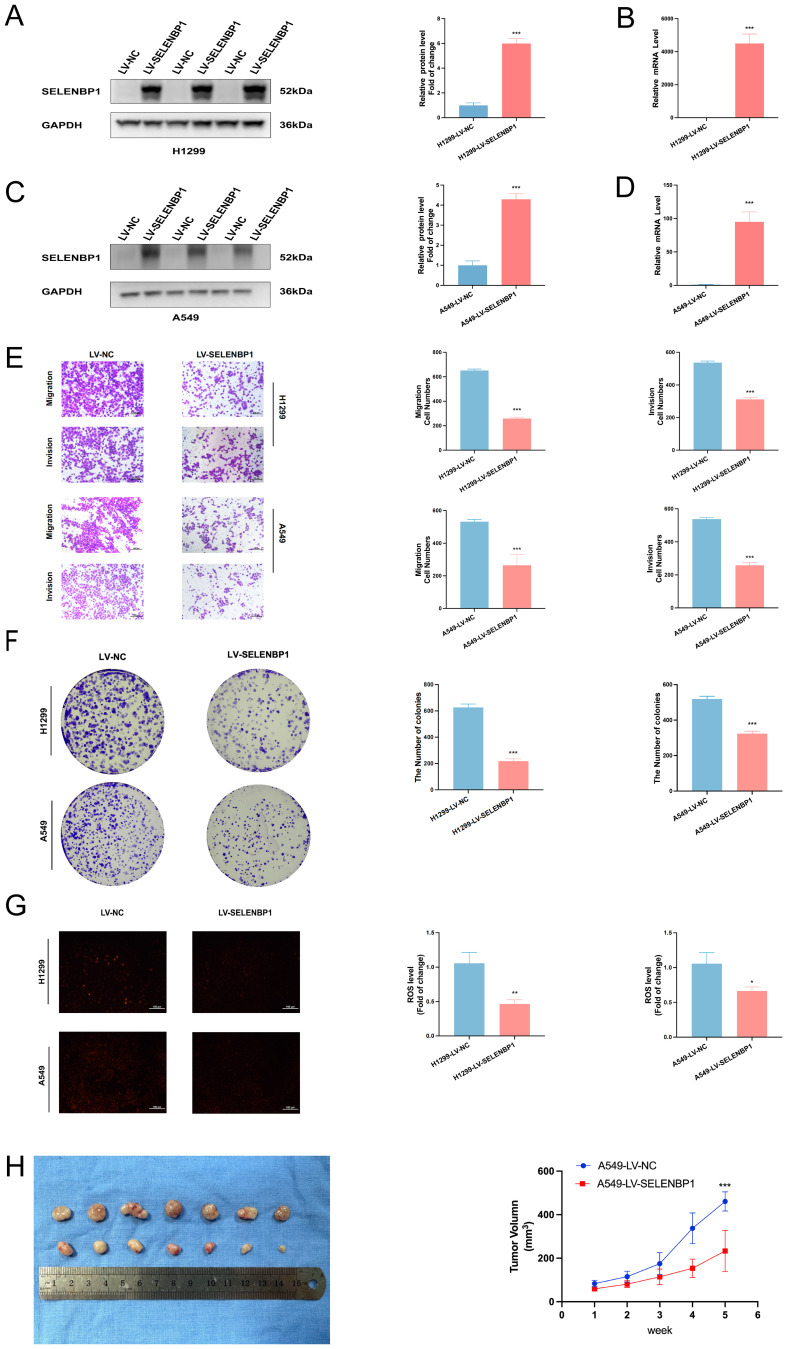
Inhibitory effects of SELENBP1 overexpression on LUAD cell migration, growth, and oxidative stress. **(A, B)** qRT-PCR and **(C, D)** Western blot confirm successful SELENBP1 overexpression. **(E)** Transwell assays demonstrate significantly reduced migration and invasion capabilities of H1299 and A549 cells following SELENBP1 overexpression. **(F)** Colony formation assays show decreased colony numbers in H1299 and A549 cells upon SELENBP1 overexpression. **(G)** ROS assays reveal significantly reduced oxidative stress levels in H1299 and A549 cells after SELENBP1 overexpression. **(H)** Nude mouse xenograft models indicate that SELENBP1 overexpression markedly suppresses tumor growth. *P < 0.05, **P < 0.01, ***P < 0.001.

Conversely, lentivirus-mediated overexpression of SELENBP1 significantly suppressed LUAD cell migratory and proliferative capacity, as shown by Transwell (**Figure 12E**) and colony formation assays (**Figure 12F**). qRT-PCR and Western blotting confirmed successful overexpression (**Figures 12A–D**). Additionally, SELENBP1 overexpression significantly reduced intracellular ROS levels (**Figure 12G**), suggesting a role in oxidative stress modulation.

To investigate the *in vivo* effects of SELENBP1, A549 cells stably overexpressing SELENBP1 were subcutaneously injected into BALB/c nude mice to establish xenograft tumors. Tumor volumes were monitored weekly. Tumors derived from SELENBP1-overexpressing cells exhibited significantly reduced growth compared to controls (**Figure 12H**), further supporting its tumor-suppressive role in LUAD.

## 4 Discussion

Lymph node metastasis in LUAD is strongly associated with poor clinical outcomes. It reflects not only enhanced tumor aggressiveness, but also increased risks of recurrence and distant dissemination, thereby serving as a critical determinant of prognosis. In recent years, advances in single-cell transcriptomics have enabled high-resolution dissection of tumor heterogeneity, reconstruction of metastatic evolutionary trajectories, and interrogation of tumor–microenvironment interactions (35). For LUAD patients with lymph node metastases, comprehensive single-cell characterization of epithelial cells within metastatic lesions provides key insights into their molecular features and developmental hierarchies. This approach facilitates the identification of metastasis- and therapy resistance–related drivers, as well as epithelial subpopulations potentially predictive of immunotherapy response (36), thereby laying the groundwork for constructing precise prognostic and therapeutic response models. Clinically, such multidimensional analyses at the single-cell level offer a transformative alternative to traditional prognostication methods, which often rely on single markers or static pathological staging. These insights enable a shift toward personalized therapeutic decision-making based on molecular subtyping and dynamic immune profiling—ultimately aiming to improve survival in high-risk LUAD patients with nodal involvement (37). Given the generally poor prognosis in this patient population, there is urgent scientific and clinical value in advancing single-cell–based investigations of epithelial heterogeneity. When coupled with large-scale clinical data integration, such efforts hold great promise for developing robust prognostic tools and guiding immunotherapy strategies, representing a pivotal step toward improving personalized outcomes in metastatic LUAD (38).

In this investigation, UMAP was employed to perform dimensionality reduction on single-cell transcriptomic datasets derived from lymph node metastases of LUAD, which facilitated the identification of four transcriptionally distinct epithelial subpopulations. Building upon this foundation, we incorporated Cox proportional hazards regression and an ensemble of machine learning methodologies to pinpoint 30 prognostic gene candidates. These core genes were subsequently integrated into the EAS scoring framework—a novel risk assessment model designed to enable refined patient stratification. The resulting EAS-based stratification system demonstrated strong capacity to distinguish LUAD patients by survival status, tumor immune landscape, mutational load, and predicted response to immunotherapeutics. Notably, patients categorized into the high-risk group exhibited significantly inferior survival outcomes compared to their low-risk counterparts. Validation across both the TCGA training cohort and multiple external GEO datasets affirmed the robust predictive performance of the model, as evaluated using receiver operating characteristic (ROC) curve analysis. Further immune profiling revealed that individuals in the low-EAS group exhibited markedly elevated expression of immune checkpoint-related and MHC-associated genes, particularly within CTLA-4–positive subsets. These features were indicative of enhanced immunological responsiveness and predicted greater clinical benefit from immune checkpoint blockade therapies. Additionally, drug sensitivity screening identified six promising small-molecule agents—Axitinib, Sinularin, BMS-754807, GSK269962A, AZD6482, and Doramapimod—with higher predicted efficacy in low-EAS patients, thus offering avenues for precision therapy development. Experimental validations using qRT-PCR confirmed the dysregulation of model genes in clinical LUAD specimens. Tumor tissues exhibited substantial upregulation of TFF1, UBE2C, ITPKA, and IGFBP3, alongside concurrent downregulation of SELENBP1 and SLC34A2, relative to matched normal samples. Functional characterization experiments further revealed that SELENBP1 functions as a tumor suppressor: its expression was consistently diminished in LUAD cell lines and tissues, and its knockdown promoted cell proliferation, migration, and resistance to apoptosis. Conversely, ectopic overexpression of SELENBP1 suppressed tumor growth by enhancing apoptosis, reducing intracellular ROS accumulation, and impairing *in vivo* xenograft progression. In summary, this study reveals the molecular heterogeneity of epithelial cells and their immune microenvironment in LUAD lymph node metastasis, establishes SELENBP1 as a potential therapeutic target, and constructs a prognostic model—EAS—based on malignant epithelial cells associated with LUAD lymph node metastasis, providing a novel theoretical foundation and application prospect for the personalized treatment of LUAD patients.

Interestingly, while high tumor mutation burden (TMB) is often associated with favorable clinical outcomes due to increased immunogenicity and enhanced response to immunotherapy, our findings revealed that a high EAS (epithelial aggressiveness score) was significantly linked to poor prognosis. This apparent contradiction underscores the complex interplay between tumor-intrinsic and tumor-extrinsic factors in shaping disease progression. TMB reflects the mutational landscape and neoantigen load of the tumor, indicating potential for immune recognition. In contrast, the EAS score captures transcriptomic features of malignant epithelial cells, emphasizing intrinsic hallmarks such as proliferative activity, plasticity, and immune evasion potential. It is plausible that in tumors with high TMB, aggressive epithelial subpopulations with high EAS may override the benefits of immunogenicity by adopting immune-suppressive or immune-resistant phenotypes. This highlights the importance of integrating both genetic and transcriptional dimensions when evaluating tumor behavior and prognostic potential, especially in the context of immunologically active microenvironments.

Our findings are broadly consistent with previous reports. Prior studies have shown that SELENBP1 is downregulated across multiple solid tumors and is frequently associated with poor prognosis. Functionally, it has been implicated in redox homeostasis and metabolic regulation, which aligns with our observations that SELENBP1 suppresses ROS accumulation, inhibits cell migration, and reduces colony formation.

In addition to SELENBP1, several other model genes identified in our study are supported by existing literature. Co-expression of TFF1 and S100P has been linked to airway dissemination in NSCLC, indicating their roles in promoting tumor progression (39). In LUAD, overexpression of UBE2C enhances ubiquitin-mediated degradation of p53, thereby attenuating the p53/p21 pathway and facilitating malignant transformation (40). ITPKA, transcriptionally activated by TFAP2A, contributes to LUAD progression through interaction with Drebrin 1 and promotion of epithelial–mesenchymal transition (EMT) (41). Elevated plasma levels of IGFBP3 have been associated with lower clinical stage, reduced Ki-67 index, and improved overall survival in lung cancer patients (42). Finally, SLC34A2 has been reported to exert a tumor-suppressive effect in NSCLC by attenuating tumorigenic potential and disease progression (43).

This study introduces several important innovations. It is the first to systematically characterize the evolutionary heterogeneity of malignant epithelial cells in LUAD lymph node metastases at single-cell resolution, enabling the construction of a trajectory-informed prognostic model. By integrating multiple machine learning algorithms, we developed an optimized EAS scoring system that balances predictive accuracy and generalizability. Moreover, the model incorporates tumor microenvironment features, tumor mutational burden (TMB), and immunotherapy response potential into a unified framework for multidimensional risk assessment. The tumor-suppressive role of SELENBP1 was also experimentally validated, providing mechanistic insights and therapeutic implications.  

Despite the comprehensive and systematic approach to data integration and mechanistic investigation, several limitations should be acknowledged. First, the majority of transcriptomic data were sourced from public databases, which may introduce sampling bias and platform-related variability. Second, although *in vitro* and *in vivo* validations were conducted, further confirmation in larger, multicenter clinical cohorts and prospective studies is necessary to fully establish the robustness and translational relevance of the EAS model. One limitation of our CNV-based analysis is the absence of an explicit doublet exclusion step prior to inference. It is recognized that epithelial–stromal or epithelial–immune doublets may introduce confounding signals and artificially alter CNV profiles, potentially affecting the accurate identification of malignant epithelial cells. To mitigate this, we applied stringent quality control filters and leveraged the built-in denoising and smoothing features of the inferCNV package, which help to attenuate noise derived from heterogeneous cell identities. Nevertheless, we acknowledge that the incorporation of dedicated doublet detection tools, such as DoubletFinder or Scrublet, would enhance the robustness of CNV-based malignant cell classification and should be considered in future analyses. Additionally, we note that the relatively dispersed pattern of CNV signals in the inferCNV heatmap may be partly due to the use of the cluster_by_groups = TRUE parameter, which enforces grouping by cell type rather than CNV similarity. While this setting improves biological interpretability, it may reduce the visual clustering of CNV patterns and should be interpreted accordingly. Additionally, predictions of immunotherapy response require validation using real-world clinical outcomes. Moreover, we observed a certain inconsistency between the pseudotime trajectory and survival analysis results. Specifically, although Cluster 1 and Cluster 2 were significantly associated with poorer prognosis (**Figure 2G**), the pseudotime trajectory inferred by Monocle2 positioned Cluster 2 at an early stage, whereas Cluster 0—which was linked to a more favorable outcome—appeared at a later stage of the trajectory. This discrepancy may be attributed to the limitations of trajectory inference algorithms, which typically rely on transcriptomic similarity to construct linear or branched trajectories. In highly heterogeneous systems like tumors, which often involve multiple evolutionary paths, such continuity-based assumptions may not accurately reflect the true temporal progression of malignant cells. In addition, cells in Cluster 2 may exhibit a “progenitor-like” or highly plastic transcriptional program, leading the algorithm to classify them as being at an earlier pseudotime point, despite their aggressive nature. In contrast, Cluster 0, although located at a later stage in the trajectory, may represent a more differentiated and transcriptionally stable subpopulation, which does not necessarily indicate higher malignancy. These findings highlight the need for cautious interpretation of pseudotime results in tumor systems and underscore the importance of integrating trajectory inference with functional and clinical outcome analyses to better understand tumor heterogeneity and progression.

Future studies may consider several key directions: (1) expanding the sample size and generating multicenter, self-derived single-cell datasets to enhance the generalizability and robustness of the findings, while including a more representative and demographically balanced patient population to reduce potential population bias;(2) conducting mechanistic investigations of the core genes included in the EAS model to uncover their regulatory networks and signaling pathways; (3) integrating real-world immunotherapy response data to assess the clinical utility of the EAS score in guiding treatment selection and monitoring efficacy; and (4) exploring the applicability of the EAS scoring system across other tumor types to lay the groundwork for a pan-cancer prognostic framework.

To further enhance the biological interpretability of the EAS model, we plan to conduct pathway-level integrative analyses of SELENBP1 and other key genes included in the model. As shown in the variable importance ranking (**Figure 4C**), other high-weight genes—such as IGFBP3, ITPKA, and UBE2C—are involved in critical oncogenic pathways, including TGF-β signaling, cell migration, and cell cycle regulation, respectively. These mechanisms may functionally antagonize or synergize with the tumor-suppressive effects represented by SELENBP1, collectively contributing to the model’s capacity to capture molecular heterogeneity. For example, previous studies have reported that IGFBP3 promotes EMT and cell invasion, whereas SELENBP1 may counteract these effects by negatively regulating ECM remodeling and suppressing TGF-β–mediated EMT processes.

## 5 Conclusion

This study elucidates the functional heterogeneity and dynamic progression of epithelial cells during lymph node metastasis in LUAD. Furthermore, it introduces a robust prognostic scoring system capable of predicting patient survival and responsiveness to immunotherapy. These findings provide a solid theoretical foundation and hold significant translational potential for risk stratification and the development of personalized therapeutic strategies in LUAD.

## Data Availability

The original contributions presented in the study are included in the article/**Supplementary Material**. Further inquiries can be directed to the corresponding authors.

## Supplementary material

Supplementary Figure 1 Quality control of single-cell data. **(A, B)** Distributions of *nFeature_RNA*, *nCount_RNA*, percentage of mitochondrial genes, and percentage of erythrocyte genes before and after quality control. **(C, D)** The distribution of cells from distal normal lung tissues and lymph node metastatic lesions before **(C)** and after **(D)** batch effect removal using the Harmony R package. **(E, F)** The distribution of malignant epithelial cell samples before (E) and after **(F)** batch effect removal using the Harmony R package.

Supplementary Figure 2 Immune microenvironment evaluation. **(A)** Comparison of immune cell composition between high and low EAS groups using seven immune infiltration inference methods. **(B, C)** Radar plots illustrating differences in immune cell populations and immune-related functions between the high- and low-risk groups. **(D)** Correlation analysis between risk score and estimate score, immune score, stromal score, and tumor purity. **(E)** Differences in stromal score, immune score, ESTIMATE score, and tumor purity between high and low EAS groups.

Supplementary Figure 3
**(A)** AUCell-based external validation of the model using the independent scRNA-seq dataset GSE149655. The t-SNE visualization shows that epithelial cells exhibit significantly higher AUCell scores than other cell types, indicating active expression of the model-involved gene set and supporting the cell-type specificity and external robustness of the model. **(B)** GSVA-based pathway enrichment analysis of the four malignant epithelial subpopulations (Cluster 0–3). The clusters showed distinct functional features: Cluster 0 was enriched in tissue differentiation and endocrine-related pathways; Cluster 1 in cell cycle pathways with high proliferative activity; Cluster 2 in inflammation, stress response, and metabolic reprogramming pathways; and Cluster 3 in developmental and stemness-related pathways, suggesting epithelial plasticity or a basal-like phenotype. **(C)** Enrichment analysis of key genes along the differentiation trajectory. Cell fate 1 was enriched in immune-related pathways such as cytoplasmic translation and granulocyte chemotaxis, whereas Cell fate 2 was enriched in pathways including positive regulation of cell adhesion, miRNA catabolic process, and heterotypic cell-cell adhesion. **(D)** Comparison of the predictive performance between the EAS model and previously published prognostic models across five independent cohorts (TCGA, GSE31210, GSE3141, GSE50081, and GSE68485). The EAS model demonstrated strong generalizability and stable prognostic predictive value across multiple independent datasets.
