# Supplementary figures and images for: Single-cell transcriptomic profiling reveals the heterogeneity of epithelial cells in lung adenocarcinoma lymph node metastasis and develops a prognostic signature
Source: Front Immunol. 2025 Jul 25;16:1637625. doi: 10.3389/fimmu.2025.1637625 (PMC12331613; doi:10.3389/fimmu.2025.1637625)

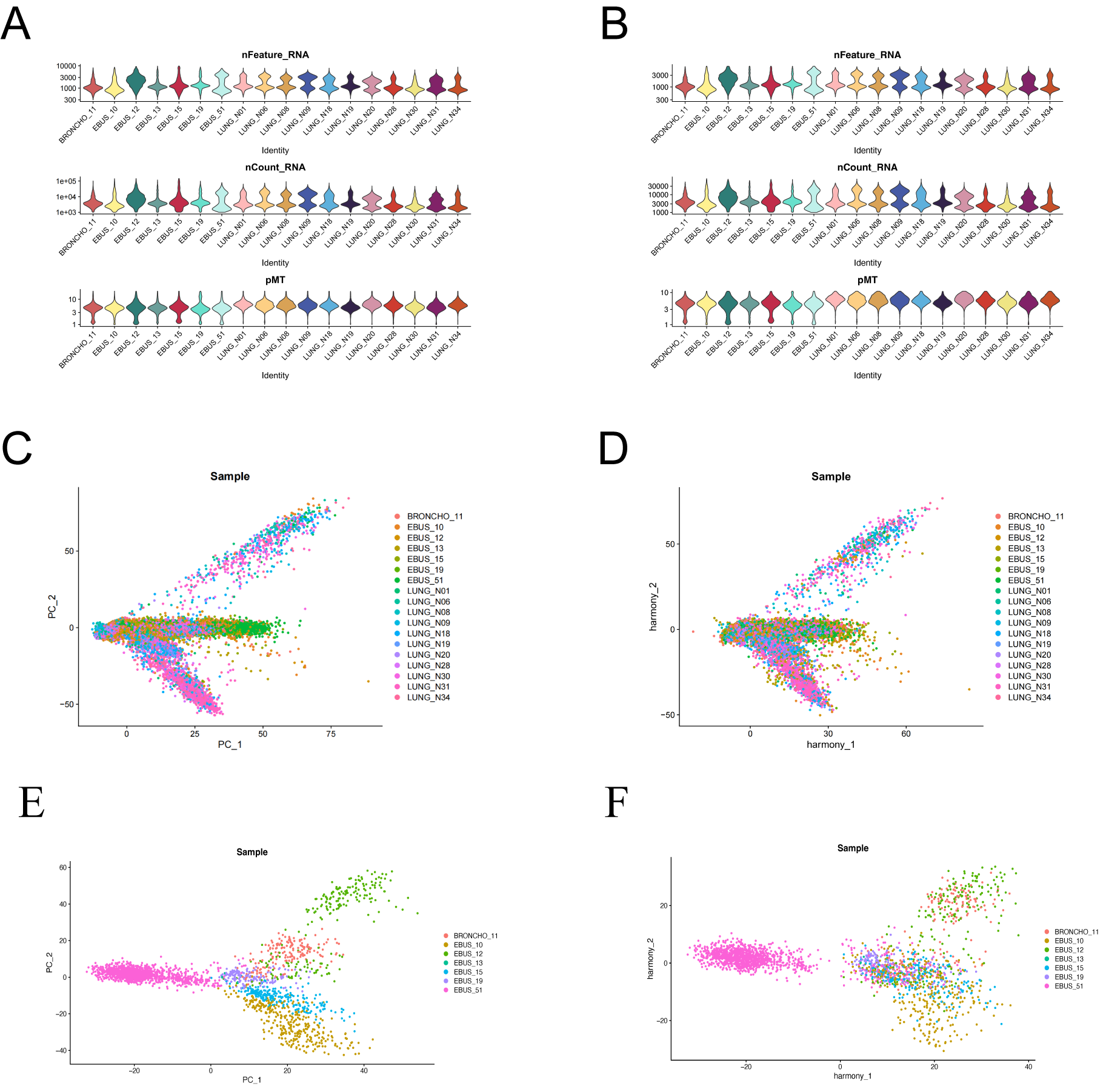

Supplement: Supplementary Figure 1 — Quality control of single-cell data. (A, B) Distributions of nFeature_RNA, nCount_RNA, percentage of mitochondrial genes, and percentage of erythrocyte genes before and after quality control. (C, D) The distribution of cells from distal normal lung tissues and lymph node metastatic lesions before (C) and after (D) batch effect removal using the Harmony R package. (E, F) The distribution of malignant epithelial cell samples before (E) and after (F) batch effect removal using the Harmony R package. [file Image1.tif]

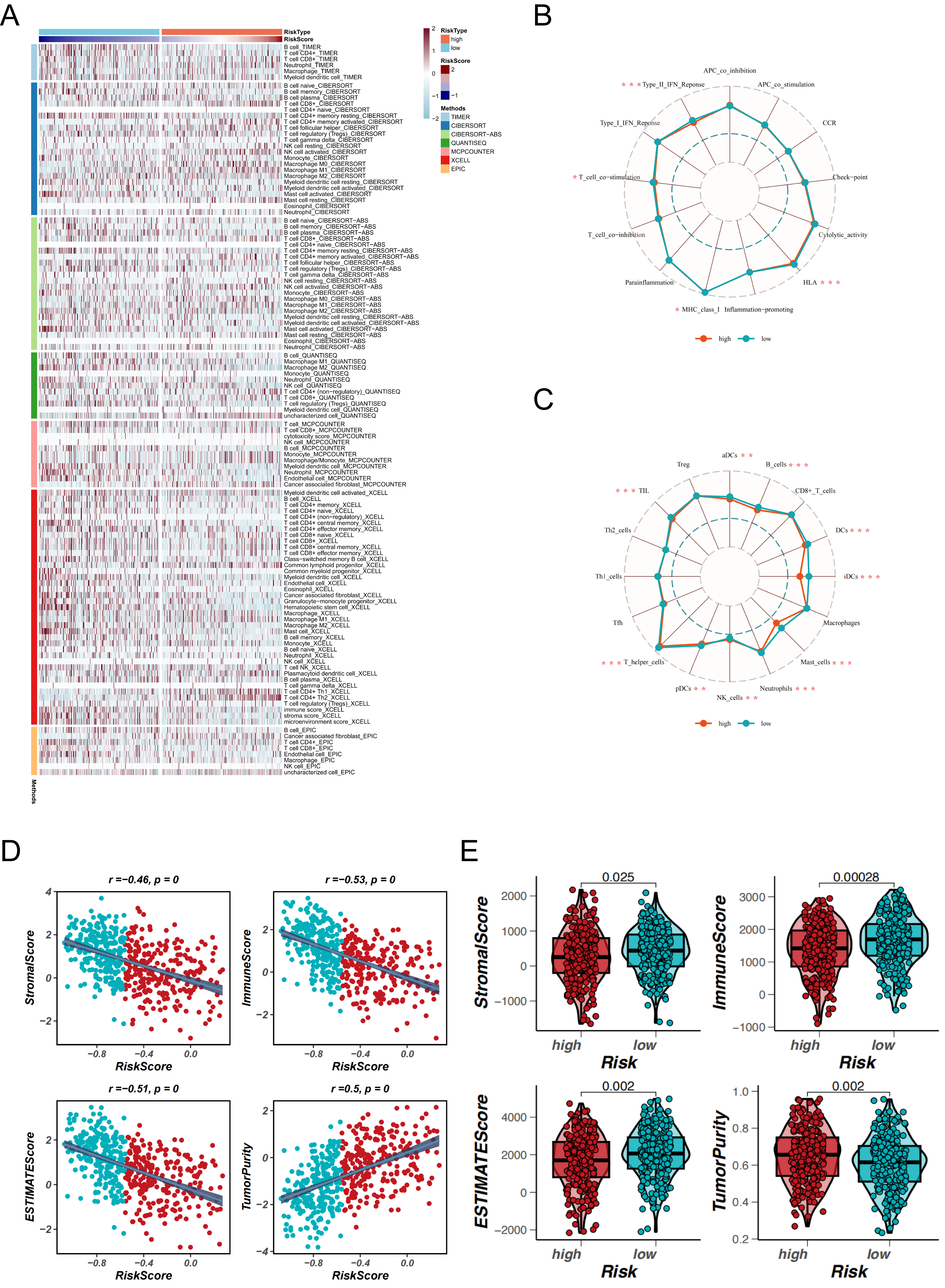

Supplement: Supplementary Figure 2 — Immune microenvironment evaluation. (A) Comparison of immune cell composition between high and low EAS groups using seven immune infiltration inference methods. (B, C) Radar plots illustrating differences in immune cell populations and immune-related functions between the high- and low-risk groups. (D) Correlation analysis between risk score and estimate score, immune score, stromal score, and tumor purity. (E) Differences in stromal score, immune score, ESTIMATE score, and tumor purity between high and low EAS groups. [file Image2.tif]

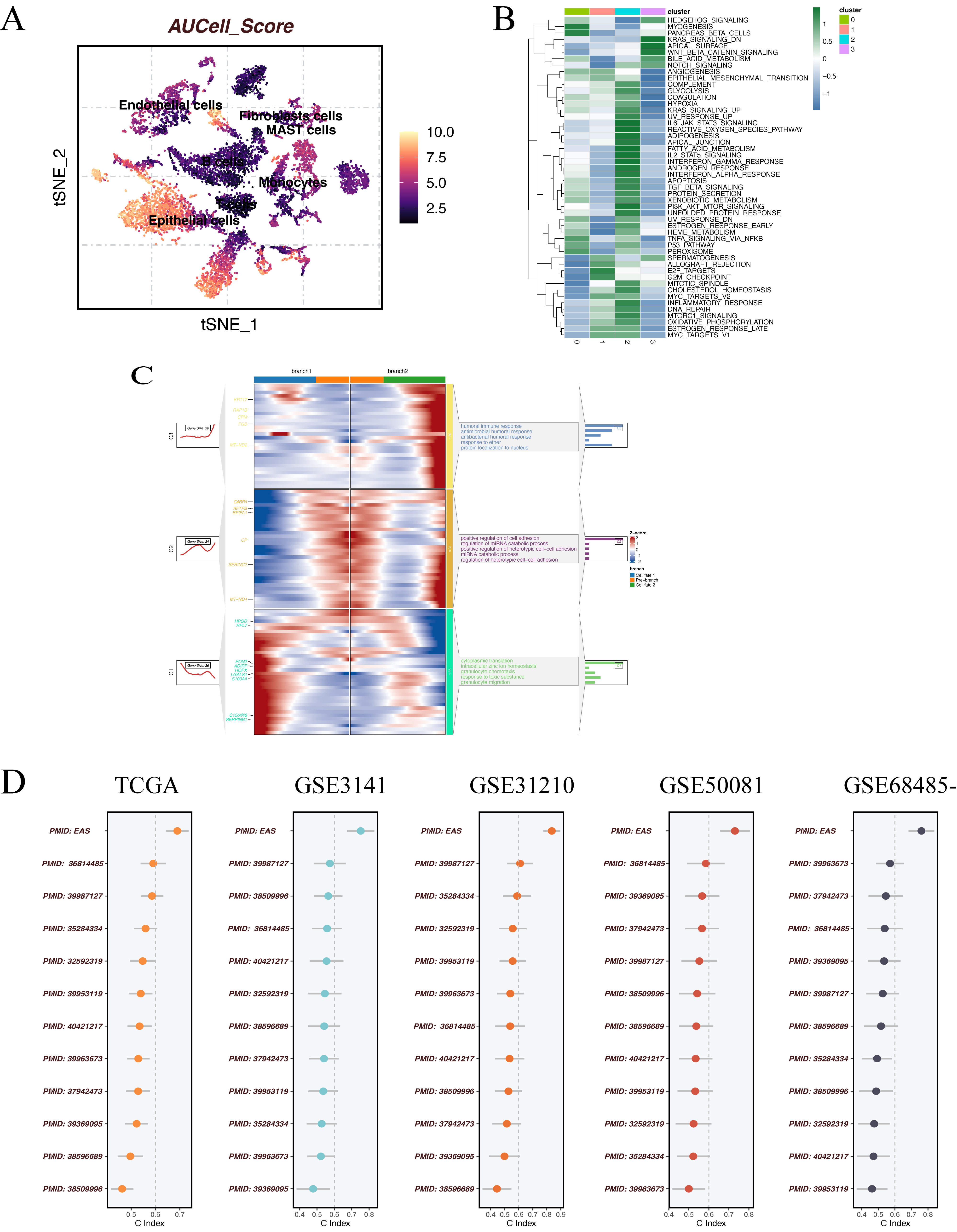

Supplement: Supplementary Figure 3 — (A) AUCell-based external validation of the model using the independent scRNA-seq dataset GSE149655. The t-SNE visualization shows that epithelial cells exhibit significantly higher AUCell scores than other cell types, indicating active expression of the model-involved gene set and supporting the cell-type specificity and external robustness of the model. (B) GSVA-based pathway enrichment analysis of the four malignant epithelial subpopulations (Cluster 0–3). The clusters showed distinct functional features: Cluster 0 was enriched in tissue differentiation and endocrine-related pathways; Cluster 1 in cell cycle pathways with high proliferative activity; Cluster 2 in inflammation, stress response, and metabolic reprogramming pathways; and Cluster 3 in developmental and stemness-related pathways, suggesting epithelial plasticity or a basal-like phenotype. (C) Enrichment analysis of key genes along the differentiation trajectory. Cell fate 1 was enriched in immune-related pathways such as cytoplasmic translation and granulocyte chemotaxis, whereas Cell fate 2 was enriched in pathways including positive regulation of cell adhesion, miRNA catabolic process, and heterotypic cell-cell adhesion. (D) Comparison of the predictive performance between the EAS model and previously published prognostic models across five independent cohorts (TCGA, GSE31210, GSE3141, GSE50081, and GSE68485). The EAS model demonstrated strong generalizability and stable prognostic predictive value across multiple independent datasets. [file Image3.tif]
